# The unity of sense and mind: A review of cross-domain mapping

**DOI:** 10.3758/s13423-025-02805-3

**Published:** 2026-01-05

**Authors:** Qiawen Liu, Gary Lupyan

**Affiliations:** 1 Department of Psychology, University of Wisconsin, Madison, WI 53706 USA; 2 Department of Computer Science, Princeton University, Princeton, NJ 08540 USA

**Keywords:** Cross-domain mapping, Metaphor, Cross-modal correspondence, Similarity

## Abstract

If the sound of a trombone had a taste, would it be bitter? In what way is solving a puzzle like navigating a relationship? People consistently map information across sensory modalities and conceptual domains. Such cross-sensory and cross-conceptual mappings have tended to be studied separately. We argue here that these mappings share underlying mechanisms and are more interconnected than previously thought. We present evidence that these mappings arise from a combination of statistical learning, magnitude matching, valence matching, and semantic mediation, involving an interplay between perception and conception. By bringing cross-sensory and cross-conceptual mappings into a common framework, we offer new insights into how people represent similarity and highlight promising avenues for understanding how humans discover and create connections across seemingly disparate domains.

## Introduction

People can easily compare things that belong to the same sensory modality or conceptual domains, such as telling whether two lights have the same brightness, whether two sounds have the same pitch, whether one event lasted longer than another, or whether the concept of doctor is more similar to that of nurse or plumber. It is thought we perform such comparisons by reporting on the representational overlap on the features/dimensions of interest (Nosofsky, 2011; Tversky, 1977). For example, tasked with comparing the brightness of two lights, we can attend to the dimension of interest—brightness—and report on its relative difference (Fotios & Cheal, 2010). What is interesting, however, is that people are *also* able to readily perform such comparisons *between* domains. We can consistently perform cross-domain mappings between different sensory modalities, such as matching visual brightness to auditory pitch (Marks, 1974, 1975, 1987; Martino & Marks, 1999; Melara, 1989) or matching auditory pitch to different tastes (Crisinel & Spence, 2010b). We can also consistently map across different conceptual domains, such as comparing space to time (Boroditsky, 2000; R. Núñez & Cooperrider, 2013; Santiago et al., 2007), space to number (Bueti & Walsh, 2009; de Hevia et al., 2014; de Hevia & Spelke, 2010; Shaki & Fischer, 2008), and musical instruments to human occupations (e.g., the concept of doctor is more similar to a piano than to a drum; Liu & Lupyan, 2023).

Cross-sensory and cross-conceptual mappings have largely developed as distinct research traditions with different theoretical frameworks*.* Cross-sensory mappings are predominantly studied in the context of multisensory integration—a process of combining noisy inputs from different modalities to form a more robust multimodal percept (Spence, 2011). To a lesser extent, they have also been studied in the context of synesthesia—a neurological condition in which stimulation of one sensory/cognitive pathway leads to automatic, involuntary experiences in a different sensory/cognitive pathway (Ramachandran & Hubbard, 2001) and iconicity—the resemblance between the form of a sign and its meaning (Dingemanse et al., 2015). Cross-conceptual mappings, on the other hand, have mostly been studied in the context of metaphors (Lakoff & Johnson, 1980a, 1999) and analogical reasoning (Gentner, 1983; Gentner & Markman, 1997).

Although cross-sensory and cross-conceptual mappings have often been investigated within their respective traditions, connections between the two types of mappings have been noted in various fields. Studies of metaphor by cognitive linguists have long emphasized the sensory basis of metaphors (Lakoff, 2009; Lakoff & Johnson, 1980b), and studies of cross-sensory mappings in language (e.g., linguistic synesthesia) can function as a type of cross-conceptual mapping (Lievers, 2017; Zhao et al., 2022). In the neuroscientific literature, it was conjectured that the same cortical regions (e.g., the angular gyrus) are involved in both metaphorical thinking and sensory synesthesia (Ramachandran & Hubbard, 2001). The purpose of this review is to more systematically examine the evidence of such correspondences which may provide insights into more general underlying mechanisms (Harnad, 2005; Shepard, 1987, 1994).

Our goal is to build on these insights by conducting a systematic comparative analysis of both types of mappings. We examine evidence from a range of empirical studies supporting the interplay between perceptual and conceptual processes in cross-domain mappings. This exploration leads to the conclusion that both cross-sensory and cross-conceptual mappings involve a synergy between relatively conceptual and perceptual levels of processing, enabling people to flexibly draw on varied types of information when making cross-domain mappings.

We begin with a brief overview of cross-sensory and cross-conceptual mappings. We then explore common mechanisms responsible for both types of mappings and discuss whether these mechanisms are best considered at a more conceptual or perceptual level. Finally, we identify outstanding questions and suggest the next steps.

## Terminology and scope

We define cross-domain mappings as correspondences between sensory modalities or conceptual domains. Within this broader domain, we use the term *cross-sensory mapping* to refer to correspondences between one sensory modality (i.e., sight, touch, smell, hearing, taste) and another sensory modality. For example, matching auditory pitch to visual brightness (Spence, 2011; Spence & Parise, 2012). Researchers have studied cross-sensory mappings between a variety of sensory modalities: *vision-audition* (see Spence, 2011, for a review), *taste/flavor-audition* (see Guedes et al., 2023; Knöferle & Spence, 2012, for reviews), *touch-audition* (e.g., Yau et al., 2009), *olfaction-audition* (e.g., Speed, Atkinson, et al., 2021a, 2021b), *vision-taste/flavor* (e.g., Motoki & Velasco, 2021), *vision-touch* (e.g., Martino & Marks, 2000) *vision-olfaction* (e.g., Demattè et al., 2009), *taste-touch* (e.g., Pramudya et al., 2020). It is likely that cross-sensory mappings exist between all conceivable domain pairs, and this body of research continues to expand. Table 1 lists 72 empirical investigations of cross-sensory mappings, showing for each study the specific domains involved and the observed associations.

**Table 1:** Selected (Although not exhaustive, the selection of studies in Table 1 and Table 2 aims to cover a wide range of cross-domain mapping, a variety of empirical tasks and populations over the past five decades, and highlighted those that provide evidence for perceptual and/or conceptual-level explanations of cross-domain mappings.) cross-sensory mappings alongside the evidence favoring perceptual and/or conceptual-level explanations. The numbers in the parentheses that follow the claim correspond to the superscript numerals in the ‘Studies’ column

Domains	Mapping	Description	Studies	Evidence favoring perceptual explanation	Evidence favoring conceptual explanation
Hearing & Sight	Pitch–size	Higher pitch to smaller, lower pitch to bigger	(Antović et al., 2020; Bonetti & Costa, 2018; Cuturi et al., 2021^1^; Eitan et al., 2014; Evans & Treisman, 2010; Fernández-Prieto et al., 2015^2^; Gallace & Spence, 2006; Mondloch & Maurer, 2004; Parise & Spence, 2009; Tonelli et al., 2017)	Visually impaired children show less pitch-size correspondence, with residual vision positively predict the strength of association (1) Observed in 6-month-old infants (2)	
	Pitch–height	Higher pitch to upper, lower pitch to lower	(Antović et al., 2020; Chiou & Rich, 2012; Dolscheid et al., 2013, 2014^1^, 2020^2^, 2023^3^; Evans & Treisman, 2010; Fernandez-Prieto et al., 2017^4^; Holler et al., 2022^5^; Korzeniowska et al., 2019^6^; Melara & Marks, 1990^7^; Melara & O’Brien, 1987; Parise et al., 2014^8^; Parkinson et al., 2012^9^; Rusconi et al., 2006; P. Walker et al., 2010^10^, 2014^11^, 2018^12^)	Observed in newborn (12), and 3 to 4 month-old infants (10–11) and 4-month-old infants across cultures (1); Observed in non-human animals like dogs (6); Large-scale natural auditory scene statistics reflect the same mapping (8); Present in populations that lack high/low labels of pitch (9)	Observed using verbal labels (*high/low)* and visual bigrams (*LO* and *HI*) (7); Language-dependent pitch-height association patterns (2–5)
	Pitch–brightness	Higher pitch to brighter, lower pitch to darker	(Anikin & Johansson, 2019; Ludwig et al., 2011^1^; Marks, 1974; Martino & Marks, 1999^2^)	Observed in chimpanzees (1)	Observed using verbal labels (*black/white)* and semantically related words (*night* and *day*) (2)
	Pitch–shape	Higher pitch to sharper, lower pitch to more round	(Marks, 1987; L. Walker et al., 2012^1^; P. Walker, 2012^2^)		Observed using pitch/shape-related words (2); Aligned on a set of semantic dimensions (1)
	Pitch–saturation	Higher pitch to more saturated color	(Anikin & Johansson, 2019)		
	Loudness–brightness	Louder to brighter in infants; flexible matching between louder to darker or louder to brighter in adulthood	(Johansson et al., 2024; Lewkowicz & Turkewitz, 1980^1^; Marks, 1974^2^; Smith & Sera, 1992^3^; J. C. Stevens & Marks, 1965^4^)	Observed in 3 to 4-week-old infants based on intensity matching (1); The matching between loudness and brightness is predicted by the psychophysical power law of sensory magnitude (4)	Adults (but not young children) can flexibly make cross-domain mapping between either loudness-brightness or loudness-dimness, depending on whether light or dark ends are treated as “more” (2–3)
	Loudness–height	Louder to upper, quieter to lower	(Bruzzi et al., 2017; Fernandez-Prieto et al., 2017; Puigcerver et al., 2019)		
	Loudness–saturation	Louder to more saturated color	(Anikin & Johansson, 2019; Whiteford et al., 2018^1^)		Explained by alignment on emotional dimensions. Partialling out the variance explained by emotion mediation eliminated the effect of correspondence between modality-specific perceptual features (1);
	Loudness–angularity	Louder to more angular, quieter to more curved	(Blazhenkova & Kumar, 2018^1^)		Observed using verbal labels (e.g., *loud, quiet)* (1)
	Tempo–saturation	Faster music to more saturated color; slower music to less saturated color	(Palmer et al., 2013^1^, 2016^2^; Whiteford et al., 2018^3^)	Cross-cultural similarity between Mexican and U.S participants (1) (though this has yet to be investigated in non-western cultures)	Explained by alignment on emotional dimensions, and the effects are highly specific to particular emotions (e.g., happy, sad, angry, calmness etc.) rather than to the generalized affective dimensions like valence and potency. (1–3) Partialling out the variance explained by emotional mediation eliminated the effect of correspondence between modality-specific perceptual features. (3);
	Tempo–hue	Faster music to yellower/warmer color; slower music to bluer/cooler color			
	Mode–saturation	Music in major mode to more saturated color; music in minor mode to less saturated color	(Palmer et al., 2013^1^, 2016^2^)		Explained by alignment on emotional mediation, and the effects are highly specific to particular emotions (e.g., happy, sad, angry, calmness etc.) rather than to the generalized affective dimensions like valence and potency. (2)
	Mode–brightness	Music in major mode to brighter color; music in minor mode to darker color			
	Tempo–brightness	Faster music to brighter color; slower music to darker color			
	Mode–hue	Music in major mode to yellower/warmer color; music in minor mode to bluer/cooler color			
	Tempo–hue	Faster music to yellower/warmer color; slower music to bluer/cooler color			
Hearing & Taste	Taste intensity–loudness	More intense taste to louder sound	(Marks, 1988^1^; Wang et al., 2016^2^)	Taste concentration – loudness is matched on intensity (1–2)	
	Taste quality–timbre	For example, bitter to brass instruments, sweet to piano	(Crisinel & Spence, 2010b, 2011^1^)	Taste-timbre are matched on valence (1)	
	Taste quality–pitch	Sweet, sour, and peppermint tastes to high pitch, umami and bitter tastes to low pitch	(Crisinel & Spence, 2009^1^, 2010a^2^, 2010b, 2011; Wang et al., 2016)		Observed using names of food items (1–2)
	Pitch–tactile size	Higher pitch to smaller tactile size	(L. Walker et al., 2012^1^; P. Walker & Smith,^ 1985^^2^)		Observed using verbal labels (*up, down, etc.)* (2)*;* Explained by alignment on a set of semantic dimensions (e.g., fast/slow, sharp/blunt, etc.) (1)
Hearing & Touch	Pitch–vibrotactile frequency	Higher pitch to higher frequency of tactile vibration	(Ro et al., 2009; Yau et al., 2009)		
	Pitch–tactile height	Higher spatial location to higher pitch	(Occelli et al., 2009)		
	Pitch–odor	Fruity/menthol odor (e.g., lemon, raspberry) to higher pitch, onion odor to low pitch	(Crisinel & Spence, 2012; Speed, Croijmans, et al., 2021a, 2021b)		
	Sound texture–tactile texture		(Bulusu & Lazar, 2024)		
Hearing & Smell	Real-world odor–sound	For example, cinnamon/orange/clove odor with Christmas carols	(Seo et al., 2014)		
Sight & Taste	Brightness–taste intensity	More intense taste (e.g., spicier) to brighter ambient environment	(Xu & Labroo, 2014)		
	Saturation–taste intensity	More intense taste to more saturated color	(Saluja & Stevenson, 2018^1^; Shermer & Levitan, 2014^2^)	Matching between valence of tastant and color (1); Correlation between magnitude of tastant concentration and color saturation (1–2)	
	Hue–taste quality	Red/pink-sweet, blue-salty, yellow-sour/sweet, green/black-bitter, white - tasteless/salty	(Saluja & Stevenson, 2018; Wan et al., 2014^1^; Woods & Spence, 2016^2^)	Cross-cultural association between bitter and black, salty and white, sour and green, sweet and pink found across China, India, Malaysia, and the U.S. participants (1)	Observed using taste/color words (e.g., sweet, yellow) (1–2)
	Hue–piqueness	Red is spicier than blue	(Shermer & Levitan, 2014)		
	Angularity–taste quality	Sweet/umami to round shapes, sour/salty/bitter/spicy to angular shapes	(Blazhenkova & Kumar, 2018^1^; Chuquichambi et al., 2024; Motoki & Velasco, 2021^2^; Seo et al., 2010^3^; Turoman et al., 2018^4^; Velasco et al., 2016^5^; Wan et al., 2014^6^)	Explained by alignment on valence and intensity (2); congruent pairs induce higher amplitudes and shorter latencies in the N1 peak of olfactory ERPs, associated with the early sensory processing of stimuli (3)	Observed using verbal labels (e.g., *sour, sweet)* (1, 5–6)*;*
Sight & Smell	Color–odor	Fragrant: e.g., floral family to warm colors and fresh family to cool colors; DKNY perfume with saturated orange and yellow, kouros with saturated blue; Natural odors: e.g., cinnamic aldehyde to red; strawberry odor to red; maple syrup odor to brown/orange	(de Valk et al., 2017^1^; Deroy et al., 2013; Gilbert et al., 1996; Goubet et al., 2018^2^; Y.-J. Kim, 2013; Demattè et al., 2006^3^; Schifferstein & Tanudjaja, 2004^4^; Speed & Majid, 2018^5^)		Explained by alignment on semantic dimensions (e.g., happy/unhappy, wild/lazy, etc.) (4); Observed using verbal labels (e.g., *strawberry odors, pink*) (3) Odor-color associations differ depending on how odors are linguistically described (1–2,5);
	Angularity–odor	For example, lemon/pepper to angular shape, raspberry/vanilla to round shape	(Hanson-Vaux et al., 2013^1^; Speed, Croijmans, et al., 2021^2^)	Angularity and odor are matched on valence and intensity (1)	Not observed in children under age 6 years (2)
Sight & Touch	Brightness–tactile size	Brighter to smaller	(L. Walker et al., 2012^1^; P. Walker & Walker, 2012^2^)		Explained by alignment on a set of semantic dimensions (fast-slow, sharp-blunt, etc.) (1–2)
	Brightness–vibrotactile frequency	Darker to low vibrotactile frequency; brighter to high vibrotactile frequency	(Martino & Marks, 2000)		
	Saturation–vibrotactile amplitude	Higher vibration amplitude to higher saturation	(T. Yuan et al., 2023^1^)	Saturation and vibration matched on intensity (1)	
	Angularity–smoothness	Curved shapes to smooth texture, angular shapes to rough texture	(Blazhenkova & Kumar, 2018^1^)		Observed using verbal labels (e.g., *smooth, rough)* (1)
Touch & Taste	Texture–taste qualities	For example, towel to sweet, linen to salty, stainless steel/rougher texture to sour, and cardboard materials to bitter	(Pramudya et al., 2020^1^; Slocombe et al., 2016^2^)	Explained by alignment on pleasantness (2)	Explained by alignment on specific emotional concept (e.g., curious, happy, peaceful, etc.) (1)
Touch & Smell	Texture–odor	smoothness to lemon/menthol odor, roughness to onion odor	(Speed et al., 2021^1^)		Associations are more likely to be observed in older age groups (1)
Smell & Taste	Odor–taste qualities	For example, sugar to sweet odor, citric-acid to sour odor	(Stevenson et al., 1999; Stevenson & Boakes, 2004)		

We use the term *cross-conceptual mapping* to refer to correspondences between elements from different conceptual domains. A conceptual domain is a set of related concepts. Such groupings of similar concepts are similar to the linguistic notion of “semantic fields” (Akmajian et al., 2001; Brinton, 2000), but while semantic fields tend to be concerned with word meanings, conceptual domains may include not only conventional superordinate categories such as animals and musical instruments, but also more schematically structured domains such as concepts related to life, relationship, and time (Lakoff & Johnson, 1999; Mandler, 1992)*.* For example, a conceptual domain of *life* encompasses a set of related concepts that include birth, death, reproduction, and growth. Cross-conceptual mappings could be established between concrete domains (e.g., physical space, human body, sensory experience) and more abstract ones (e.g., time, numbers, emotions, valenced concepts, and power). For example, representations of words related to time, like “before” and “after,” or durations like “short” and “long” appear to have a spatial component (see Bender & Beller, 2014; R. Núñez & Cooperrider, 2013, for a review). Evidence for this spatial component comes from phenomena such as the Spatial-Temporal Association of Response Codes (STEARC effect), where people’s responses to past or short-term events are faster with the left hand, and future or long-term events are faster with the right hand (Anelli et al., 2018; Ishihara et al., 2008; Santiago et al., 2007; Vallesi et al., 2008). Similar patterns of spatialization are also seen for numerical concepts as reflected in Spatial-Numerical Association of Response Codes (SNARC effect), where people are faster to respond to relatively small numbers with their left-hand side and faster to relatively large numbers with their right-hand side (see Fischer & Shaki, 2014; Toomarian & Hubbard, 2018, for reviews). Table 2 lists 84 empirical investigations of cross-conceptual mappings, showing for each study the specific domains involved and the observed associations.

**Table 2:** Selected cross-conceptual mappings and the literature, and evidence favoring perceptual/conceptual explanation. The number in parentheses following each evidence corresponds to the superscript in the ‘Studies’ column

Domains	Mapping	Description	Studies	Evidence favoring perceptual explanation	Evidence favoring conceptual explanation
Power and Space	Powerfulness–verticality	Powerful is spatially upper, powerless is spatially lower	(Dahl & Adachi, 2013^1^; Giessner et al., 2011^2^; Giessner & Schubert, 2007^3^; Meier & Dionne, 2009; Niedeggen et al., 2017; Schoel et al., 2014; Schubert, 2005^4^)	Observed in chimpanzees (1);	Observed using linguistic stimuli (e.g., *master, servant, leader,* etc., 2–4);
Valence and Space	Valence–verticality	Positive is spatially upper, negative is spatially lower	(Crawford et al., 2006; Gottwald et al., 2015; Lakens, 2012^1^; Lakens et al., 2012^2^; Lynott & Coventry, 2014^3^; B. Meier et al., 2004^4^; B. P. Meier, Hauser, et al., 2007^5^; B. P. Meier, Sellbom, et al., 2007^6^; B. P. Meier & Fetterman, 2022^7^)		Observed using linguistic stimuli (e.g., *love, hate, etc.*, 4–7); The correspondence between +polars are more automatic and the automaticity of this correspondence could be diminished by reversing the frequency of polar during experiment (1–3)
	Valence–horizontality	Positive things are associated with people’s dominant side of space and negative emotion is associated with their non-dominant side of space; more intense emotion is mapped onto more right side of space	(Casasanto, 2009^1^; Casasanto & Chrysikou, 2011^2^; de la Vega et al., 2013^3^; Holmes & Lourenco, 2011; Pitt & Casasanto, 2018^4^)	Left-handers associate left-hand side space with positive valence, despite the right-is-good coding in language; (1); By temporarily handicapping one’s dominant hand, participants’ valence-horizontality (association could be reversed (2)	Observed using linguistic stimuli (3–4)
Valence and Brightness	Valence–brightness	Good is bright, bad is dark, and vice versa	(B. Meier et al., 2004^1^; B. Meier, Robinson, et al., 2007^2^; Okubo & Ishikawa, 2011^3^; Sherman & Clore, 2009; Song et al., 2012^4^; Xu & Labroo, 2014^5^)		Observed using linguistic stimuli (e.g.*, gentle, devil*, etc., 1–5)
Time and Space	Ego/object moving in time–ego/object moving in space	People primed by ego/object moving have consistent preference for ego/time moving interpretation of an ambiguous sentence, but primed by ego/time moving does not lead to consistent preference for spatial ego/object moving	(Boroditsky, 2000^1^; Boroditsky & Ramscar, 2002^2^)		Observed using linguistic stimuli/measure (e.g., ‘The meeting originally scheduled for next Wednesday has been moved forward two days’ – the meeting will be on Monday/Friday?) Primed by ego/object moving have consistent preference for ego/time moving interpretation of an ambiguous sentence. Primed by ego/time moving doesn’t lead to consistent preference for spatial ego/object moving, suggesting an independent conceptualization of time without necessary activation of space domain (1–2)
	Duration–spatial displacement	Spatial displacement–estimates of duration	(Athanasopoulos & Bylund, 2023^1^; Boroditsky, 2008^2^; Bottini & Casasanto, 2013^3^; Bylund & Athanasopoulos, 2017^4^; Casasanto & Boroditsky, 2008^5^; Gijssels et al., 2013^6^, 2013^7^; Lourenco & Longo, 2011^8^; Merritt et al., 2010^9^; Srinivasan & Carey, 2010^10^)	Observed in preverbal infants and non-human animals (symmetric mapping between space and time: incongruent spatial displacement influence estimates of duration; incongruent duration influence estimates of spatial displacement) (8–10)	Asymmetrical relationship between duration and spatial displacement in human adults and children with language ability (3–7); People speaking different languages and bilingual people’s estimation of duration are affected differently by language used in context, which has different spatial metaphors for duration (1,4)
	temporal progression–spatial direction	Time moves along spatial direction such as left-right, up-down, uphill-downhill, east-west	(Boroditsky et al., 2011^1^; Fuhrman et al., 2011^2^; Fuhrman & Boroditsky, 2010^3^; R. E. Núñez & Sweetser, 2006^4^; Santiago & Lakens, 2015^5^)		People speaking languages with different spatial metaphors of temporal progression have different conceptualization for temporal progression (1–5)
Number and Space	numerical scale–vertical/horizontal line	Large numbers are associated with bottom/right decision and small numbers are associated with top/left decision	(Bulf et al., 2016^1^; de Hevia et al., 2014^2^; Dehaene et al., 1993; Drucker & Brannon, 2014^3^; Fischer & Shaki, 2014; Ito & Hatta, 2004^4^; Rugani et al., 2015; Santiago & Lakens, 2015; Shaki & Fischer, 2008^5^; Zohar-Shai et al., 2017^6^)	Observed in nonlinguistic infants (2) and nonhuman animals (1,3)	Whether people show left/right or top/bottom association of number depends on the writing system they use (4–6)
	ordinality of numbers–letters to temporal progression	People think about ascending numbers/letters have consistent preference for ego moving interpretation of an ambiguous sentence	(Matlock et al., 2011^1^)		Observed using linguistic stimuli/measure (e.g., ‘The meeting originally scheduled for next Wednesday has been moved forward two days’ – the meeting will be on Monday/Friday) (1)
Morality and Physical Cleanness	Morality–purity	Moral contamination is mapped to physical contamination	(Lee & Schwarz, 2010^1^; Schnall et al., 2008^2^; Stellar & Willer, 2014^3^; Zhong & Liljenquist, 2006)		Observed using linguistic stimuli/measure(e.g., lying, moral judgment, read a story, 1–3)
Affection and Warmth	Emotion/social proximity–temperature	Experiencing physical warmth/coldness induce social affiliation/loneliness and vice versa	(Blumberg et al., 1992^1^; Harlow, 1958^2^; IJzerman & Semin, 2009^3^; Inagaki & Eisenberger, 2013^4^; Schilder et al., 2014^5^; Williams & Bargh, 2008a; Zhong & Leonardelli, 2008)	Observed in nonhuman animals (1–2); social warmth and physical warmth share neural mechanism associated with warmth and rewarding outcomes (4)	Observed using linguistic stimuli/measure (e.g., describe feeling with language) (3–5)
Emotional distance and Physical distance	Emotion/social proximity–physical proximity	Judgments of the strength of their emotional attachments to important aspects of their social world are enhanced/reduced given large/small physical-distance cues	(Williams & Bargh, 2008b^1^)		Observed using linguistic stimuli/measure (e.g., read a story and rate how much they like it) (1)
Importance and Weight	Social importance–physical weight	Judgment of importance are higher when accompanied by larger physical weight	(Jostmann et al., 2009^1^)		Observed using linguistic stimuli/measure (e.g., read about a public issue and rate how much people think their voice matter (1))
A mix of conceptual domains	Predicate metaphors	A motor verb is used to act on intangible things	(Boulenger et al., 2009^1^; Desai et al., 2013^2^; Wilson & Gibbs, 2007^3^)	Observed neural activation in motor cortex when reading the predicate metaphors (2)	Observed using linguistic stimuli (e.g., *grasp,*1–3*)*
	Adjective metaphors	An adjective initially used to describe one domain is used to describe another domain	(Citron & Goldberg, 2014^1^; Lacey et al., 2012^2^)	Observed neural activation in sensory cortex when reading the adjective metaphors (1–2)	
	Nominal metaphors	A is B while A and B are from disparate semantic domains	(Al-Azary & Katz, 2021^1^; Liu & Lupyan, 2023^2^; Tourangeau & Sternberg, 1981^3^, (Tourangeau, et al., 1982)^4^; Zhu et al., 2024^5^)	When metaphors are novel, it activates the embodied feature of the source domain (e.g., after reading *my lawyer is a shark,* people’s response to embodied feature of shark, e.g., *bite* may be boosted due to embodied simulation) (1)	Observed using linguistic stimuli (1–5); Explained by alignment on a set of semantic dimensions (fast-slow, sharp-blunt, etc.) (2–4)
	Body-related metaphors	There’s non-arbitrary connection between body parts and symbolic meanings across cultures	(Holmes et al., 2018^1^)	e.g., ‘idea’ in Chinese can be literally translated as ‘heart cut’, and non-Chinese speakers have non arbitrary intuitions about these compound words (1)	Observed using linguistic stimuli (1)
	Discourse-level metaphor	Discourse-level metaphorical framing impacts people’s perception of the target domain.	(Flusberg et al., 2018^1^; Hendricks et al., 2018^2^; Thibodeau & Boroditsky, 2011^3^)		Observed using linguistic stimuli (e.g., read a story about crime in a city, 1–3)
	Relational mappings	People map across two patterns based on relational structure rather than object attributes	(Casasola, 2005^1^; Christie & Gentner, 2014^2^; Gentner et al., 2013^3^; Loewenstein & Gentner, 2005^4^; Simms & Gentner, 2019^5^)		Relational language facilitates abstraction of relational information (1–5)

To better isolate generalizable mechanisms, we will focus on studies conducted on healthy, neurotypical individuals, omitting work on individuals with neurological impairments (e.g., focal lesion: Cardillo et al., 2018; neurodegeneration: Klooster et al., 2020; Parkinson: Monetta & Pell, 2007; Alzheimer: Papagno, 2001) and traits like synesthesia (e.g., Hubbard et al., 2005; Ramachandran & Brang, 2008; Ramachandran & Hubbard, 2001). We also omit work focusing on iconicity and sound symbolism (Delgado et al., 2020; Emmorey, 2014; McCormick et al., 2021; Ozturk et al., 2013; Strickland et al., 2015, 2017) despite the likely convergence between these phenomena and those underlying cross-sensory/cross-conceptual mappings (Perniss et al., 2010; Sidhu & Pexman, 2018; Westbury et al., 2018).

## Overview of cross-sensory and cross-conceptual mappings

### Experimental paradigms for studying cross-domain mappings

**Fig. 1:**
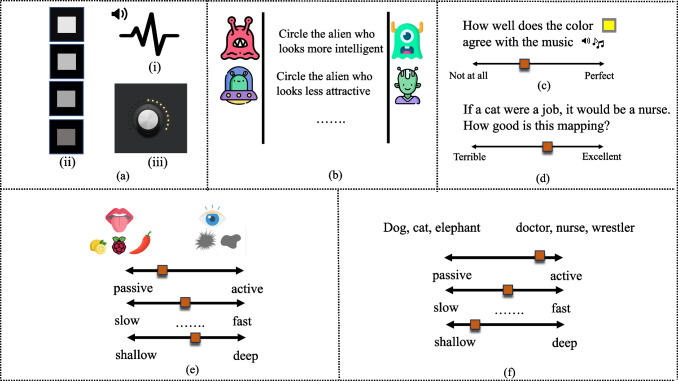
Schematic illustration of tasks for evaluating people’s judgment of compatibility between cross-domain items. **(a)** A matching task for cross-sensory (in this case, pitch-lightness) mapping. **(b)** A matching task for cross-conceptual (e.g., valence-space) mapping, participants are asked to choose whether the alien on the left-hand or right-hand side is more positive/negative. **(c)-(d)** Participants indicate how well they think two stimuli go with each other using a Likert scale **(e)-(f)** Participants rate a range of cross-sensory (e.g., taste and shape) or cross-conceptual (animals and jobs) on a set of dimensions. (Color figure online)

The most direct approach to probe cross-domain mappings is to have participants evaluate the compatibility of specific pairings. For instance, researchers might ask participants to assess whether a high pitch better goes with a bright or a dim light. This can be done explicitly by using a matching task: present participants with stimuli from one domain and then ask them to choose which of several options from another domain is best aligned (e.g., Wang & Spence, 2017). Another task is to present participants with a perceptual stimulus (a color, a shape, a piece of music, etc.), or a word and ask them to rate it on a series of scales anchored by pairs of opposites (e.g., *good/bad, high/low, slow/fast*; i.e., the so-called semantic differential technique; Osgood et al., 1957). This allows researchers to evaluate the overall similarity between items from different sensory or conceptual domains within a common space (e.g., Liu & Lupyan, 2023; L. Walker et al., 2012; Whiteford et al., 2018). Figure 1 shows some of these tasks.

**Fig. 2:**
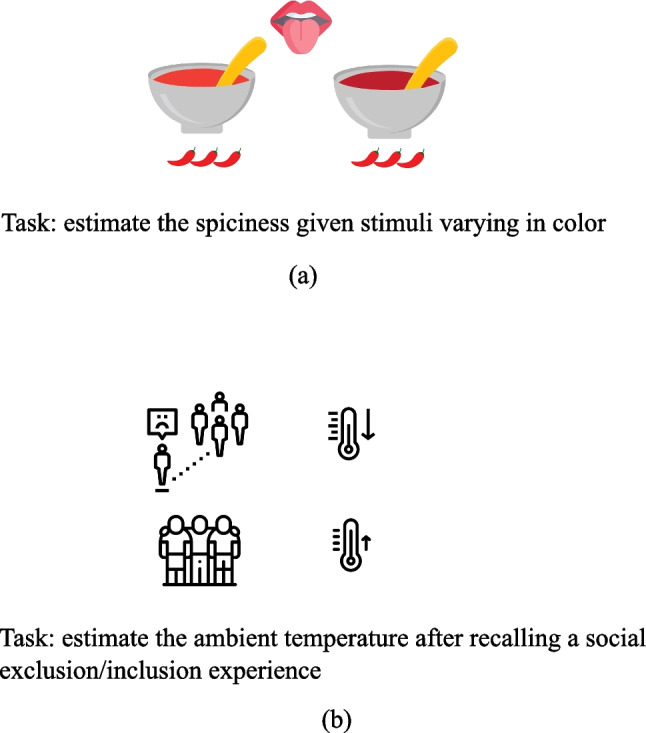
Manipulating stimuli from one domain impacts participants’ experiences in another domain. **(a)** Participants rated the darker salsa as spicier. **(b)** Participants estimated the same ambient temperature as lower after recalling a socially exclusionary experience compared to recalling a socially inclusive experience. (Color figure online)

However, these tasks are inherently subjective and open-ended, limiting the conclusions one can draw about the cognitive and perceptual consequences of a given mapping. An alternative approach is to establish an objective ground truth and observe how manipulating stimuli from one domain impacts participants’ experiences in another domain. For example, in a study by Shermer and Levitan (2014), participants rated the spiciness of salsas that varied in color and piquancy. The results showed that spiciness ratings were influenced by color: darker salsa was rated as spicier than lighter salsa despite having the same objective level of spice. Similarly, people estimated the same ambient temperature to be lower after recalling a socially exclusionary experience compared to recalling a socially inclusive experience (Zhong & Leonardelli, 2008; Fig. 2).

**Fig. 3:**
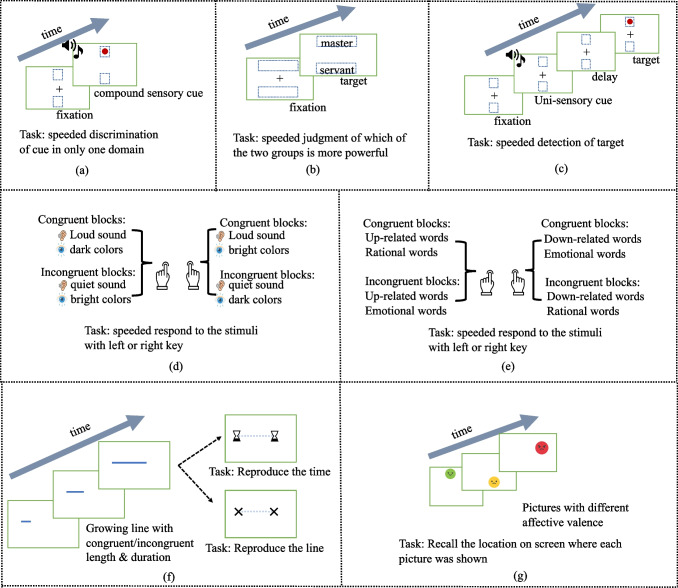
**(a)** People are faster to classify a visual stimulus on the up/down side of the screen when hearing higher/lower pitch. **(b)** People are faster to detect visual targets in upper (or lower) spatial locations while hearing a higher (or lower) pitch sound. **(c)** People are faster to classify which group is more powerful when the more powerful group is displayed on the vertical position on the screen. **(d)-(e)** People match cross-sensory stimuli (e.g., sound and colors) or cross-conceptual stimuli (e.g., verticality-related words and emotional/rational words) with two response keys, and only one stimulus is present at a time. If the congruent stimuli (e.g., loud sound and dark color, up-related words and rational words) match the same key, they respond faster and more accurately than when they are incongruent. **(f)** Participants were shown computer-generated animations of a line growing over time and were asked to reproduce the duration or reproduce the time. They tended to overestimate time when the line was long and underestimate it when the line was short. **(g)** Participants were shown valenced pictures on different vertical locations, afterwards they recalled positive images as appearing in higher locations relative to negative images, reflecting a “GOOD is UP” mapping. (Color figure online)

Another layer of subjectivity in such tasks is response bias. For example, participants may alter their response patterns to be more in line with what they believe the experimenter expects (Firestone & Scholl, 2014; Zizzo, 2010). Consequently, it remains unclear whether any consistent mappings revealed in the above ways are the result of representational overlap across domains or participants simply responding in the way they think they are expected to respond. Alternatively, researchers have used tasks that are designed to elicit more automatic, less consciously controlled responses. One such task is speeded discrimination, where participants need to classify stimulus features from one sensory domain on a directional scale (e.g., small-large, low-high) as fast as possible, while there’s a completely task-irrelevant stimulus in another sensory domain present that has either congruent or incongruent cross-sensory-domain directional features (e.g., Marks, 1987; Schubert, 2005). If congruent stimuli lead to faster, more accurate responses (i.e., congruence effect), it lends support to the automaticity of the cross-domain mapping (Fig. 3a–b). Other paradigms intended to test such automatic responses include speeded detection (e.g., Chiou & Rich, 2012, Fig. 3c) and the Implicit Association Test (IAT, e.g., Anikin & Johansson, 2019; Fig. 3d–e). Memory tasks offer another approach to measure the objectivity of cross-domain mappings, as the participants’ ability to accurately recall or reproduce information is not likely to be consciously controlled (Casasanto & Boroditsky, 2008; Crawford et al., 2006; Fig. 3f–g). For example, to investigate the mapping between time duration and spatial length, participants were shown computer-generated animations of a line growing over time and were asked to reproduce the duration. The length of the lines was irrelevant to the task of duration estimation. However, people could not disregard incompatible spatial information when reproducing time durations, as they tended to overestimate time when the line was long and underestimate it when the line was short (Casasanto & Boroditsky, 2008; see also Bylund & Athanasopoulos, 2017, for an analogous task probing duration-size mapping, where participants were asked to reproduce duration after seeing an animation of a container being filled gradually with liquid).

### What makes cross-domain mappings possible?

Evidence of cross-domain mappings goes beyond tasks requiring simply matching stimuli across domains, extending to tasks thought to tap into more automatic processes, with tell-tale signs of facilitation and impairments in accuracy and RTs. But why?

Spence (2011) discusses three mechanisms underlying cross-sensory mappings. *Structural correspondence* results from common neural coding across modalities. For example, loud sounds may map onto bright lights because both are coded through increases in neural firing rate. *Statistical correspondence* results from repeated co-occurrences (e.g., a mapping between small size and high pitch can arise from experiences associated with hearing objects fall: small objects ping; large objects thud). *Linguistic correspondence* arises from the use of the same words or phrases across domains, such as when “high” and “low” are used for both height and pitch. Importantly, these mechanisms are not mutually exclusive. For example, the cross-sensory mapping between pitch and elevation can be explained by both statistical correspondence and linguistic correspondence. More recent works introduced other explanations such as *valence matching*—where sensations are aligned based on the similarity of their emotional appeal or repulsion (Motoki et al., 2022; Saluja & Stevenson, 2018), emotion mediation—which aligns sensations based on shared emotion content (Palmer et al., 2013; Salgado Montejo et al., 2015; Schifferstein & Tanudjaja, 2004), and semantic mediation—which align sensations based on shared semantic meanings (Velasco et al., 2016; P. Walker, 2012, 2016)

Similarly, several mechanisms have been proposed to explain cross-conceptual mappings. Conceptual metaphor theory (Lakoff & Johnson, 1980a, 1980b) suggests that we understand abstract concepts via concrete experiences through embodied simulation. A theory of magnitude (ATOM; Walsh, 2003) suggests there is a domain-general magnitude system responsible for processing different quantities and enabling comprehension of ‘more’ or ‘less’ across various domains. The semantic mediation account (Dolscheid et al., 2013; Lakens, 2012; Liu & Lupyan, 2023) suggests that the similarities in the way things are represented in an abstract semantic space, lexicalized, or shaped by cultural-linguistic factors such as linguistic markedness, may systematically connect two conceptual domains. Again, these mechanisms are not mutually exclusive; the same phenomenon, such as mapping between space and time, could be explained by experiential correlations between space and time, the domain-general magnitude system, and the linguistic mechanism of using spatial terms to describe time.

It is already evident that the proposed mechanisms of how people make cross-sensory and cross-conceptual associations are very similar. In the following section, we sought to organize these mechanisms into four categories: first, cross-domain mappings emerge from statistical learning from the natural environment; second, cross-domain mappings are matched by magnitude; third, cross-domain mappings are matched by valence; fourth, cross-domain mappings are mediated by semantics. As we will argue, these non-mutually exclusive mechanisms provide a robust framework for understanding how humans make reliable mappings across domains.

## Common mechanisms of cross-sensory and cross-conceptual mappings

### Statistical learning from the natural environment

Our experiences of the world around us are inherently multisensory. For example, when we eat, we not only taste the food but also perceive its smell, texture, temperature, and appearance. The frequency with which two stimuli occur together can strengthen the activated neural connections, leading to the formation of cross-sensory mappings, a core principle of learning through association, often summarized by the phrase “cells that fire together, wire together” (Hebb, 1949).

Some universal (or near-universal) cross-conceptual mappings are believed to be grounded in such experiential correlations. One example is the mapping between time and space as reflected in both language and thought (a “long walk” takes more time than a “short” walk, while in general, traversing a longer distance; Casasanto & Boroditsky, 2008). Observing natural phenomena, such as the sun’s journey from east to west and the progression from dawn to dusk also accompanies visual-spatial changes in our surroundings with the passage of time (Boroditsky & Gaby, 2010). Specifically, statistical learning from the natural environment explains cross-domain mappings found widely in various species. Macaque monkeys, for instance, associate vocal tract resonances with visual size, matching large-sounding coos to larger faces and small-sounding coos to smaller faces (Ghazanfar et al., 2007).

The role of statistical learning in shaping cross-domain mappings might be underestimated. Studies that identify consistent cross-domain mappings often disregard statistical learning as an explanation, citing the absence of corresponding real-world regularities. For example, statistical learning was ruled out as an explanation for brightness–pitch mappings because bright objects do not inherently produce higher-pitched sounds than dark objects (Ludwig et al., 2011). However, such associations do occur. For example, alarms that pair loud high-pitch sounds with bright flashing lights. Even if such first-order associations were entirely absent from a learner’s experience, statistical learning could be based on higher-order associations (possibly first described by William James, 1890, as the law of dissociation by varying concomitants; see also Fiser & Aslin, 2002a, 2002b; Tighe & Tighe, 1966). Brightness and high pitch can become conjoined if both occur in similar contexts (e.g., both are used as effective attention-getters even if never at the same time). Furthermore, crossmodal correspondences can be transitive. Thus, the scope of statistical learning in shaping cross-domain mappings likely includes both first-order and higher-order associations, though the exact contribution of different orders remains to be empirically investigated.

Statistical learning, however, may not always be an adequate explanation of cross-domain mappings. First, some cross-domain mappings are observed even in neonates who have extremely limited experience with the relevant first- or higher-order associations. For instance, neonates aged 0–3 days showed prolonged attention to simultaneous increases (or decreases) in spatial extent, duration, or numerical quantity, but not when these dimensions varied in opposite directions (one increased while the other decreased; de Hevia et al., 2014). Another challenge to statistical learning as an explanation is structural cross-domain mappings such as understanding the structure of an atom by drawing insight from the structures of a blueberry muffin, a solar system, or a cloud. In these cases, cross-domain mappings might require processes like analogical reasoning, which transfer relational structures from one domain to another (Gentner, 1983).

### Magnitude matching

Another explanation for cross-domain mappings is magnitude-matching: matching more to more, and less to less. One example is Walsh’s (2003) ATOM, which seeks to explain how people represent fundamental domains of experience such as length, area, numerosity, temporal duration through a generalized magnitude system. ATOM is supported by observed behavioral interference between cross-domain magnitude dimensions (Bueti & Walsh, 2009; Lindemann et al., 2008). Interference and congruence effect between different magnitudes is found to be modulated by the activity in the parietal lobe—the neuronal substrate proposed for domain-general magnitude processing (Belin et al., 1998; Bueti & Walsh, 2009; Cohen Kadosh & Walsh, 2009).

The generalized magnitude system is proposed to be involved not only when matching along properties such as size, distance, and duration, but also the intensity of various stimuli (Cohen Kadosh et al., 2008; Hartmann & Mast, 2017; Lindemann et al., 2008; Pinel et al., 2004; Vierck & Kiesel, 2010). The relationship between intensity of stimulus and magnitude of sensation was explored by S. S. Stevens (1957), who proposed two types of sensory continua. *Prothetic* continua differ in quantity, while metathetic continua differ in quality. For instance, the emotions of happiness and ecstasy differ in intensity (prothetic), whereas happiness and sadness differ in type (metathetic). Stevens demonstrated that the relationship between stimulus intensity and sensation magnitude for prothetic dimensions can be mathematically expressed using what is generally known as Stevens’ power law. Magnitude matching therefore also explains cross-domain mapping with similar levels of arousal or excitement. For example, loud sounds and bright colors have a similarly high level of excitation of their sensory domains (Marks, 1974, 1987; J. C. Stevens & Marks, 1965); auditory loudness maps onto the intensity of a gustatory stimulus (Smith & Sera, 1992), color saturation is mapped onto tastant concentration (Saluja & Stevenson, 2018; Shermer & Levitan, 2014).

Magnitude matching has been proposed as one way to explain cross-domain mappings evident in the earliest days of life. It is nonverbal and operational from birth or early infancy, before the development of higher-level conceptual abilities (de Hevia et al., 2014; Lewkowicz & Turkewitz, 1980; Mondloch & Maurer, 2004; P. Walker et al., 2014) and is a shared trait between humans and animals, with non-human primates and birds also demonstrating sensitivity to analogous magnitude relations (Adachi, 2014; Drucker & Brannon, 2014; Ghazanfar & Maier, 2009; Ludwig et al., 2011; Merritt et al., 2010; Rugani et al., 2010, 2015). However, magnitude matching struggles to fully explain mappings between more complex stimuli. This includes mappings involving metathetic dimensions without clear magnitude arrangement (e.g., hue and taste). Similarly, when it comes to stimuli varying along multiple dimensions, such as matching music with colors, only a fraction of dimensions—like loudness of sound or color saturation are magnitude-relevant. Additionally, magnitude matching alone doesn’t easily account for mappings involving abstract symbols like numbers, which lack inherent perceptual hierarchies of more or less.

### Valence matching

Evaluating valence is a basic ability already present in infancy (Quinn et al., 2011; Ruba et al., 2019; Steiner et al., 2001) and observed widely across the animal kingdom (Berridge & Kringelbach, 2015), suggesting that it serves an important role in helping organisms assess potential threats. Interestingly, people automatically evaluate the valence not only of clearly valenced stimuli, such as guns or roses, but also of objects often considered neutral in everyday contexts, such as mugs or ketchup (Bradley & Lang, 1999; Lebrecht et al., 2012). Valence matching is proposed to explain cross-domain stimuli based on a common evaluative or hedonic system, which is underpinned by neural activity not only in multimodal regions known for processing emotion content, such as the amygdala and orbitofrontal cortex (LeDoux, 2003), but also within the primary sensorimotor cortices (Bestelmeyer et al., 2017; Gao & Shinkareva, 2021; Miskovic & Anderson, 2018; Sievers et al., 2013, 2021).

Cross-domain mappings based on valence matching—"good” stimuli in one domain go with “good” stimuli in another domain—have been proposed to explain consistency in mapping tastes to music genres (e.g., Motoki et al., 2022), timbres (e.g., Crisinel & Spence, 2010b), colors (e.g., Saluja & Stevenson, 2018), and pitches (e.g., Wang et al., 2016), as well as associating odors with colors (e.g., Y.-J. Kim, 2008). For example, the association between sweetness and the color pink may arise from both being linked to pleasantness whereas bitterness and blackness might both be linked with negative valence (Palmer & Schloss, 2010; Spence & Levitan, 2021).

Beyond cross-sensory mappings, valence matching also provides insights into cross-conceptual mappings, particularly when perceptions of good or bad in more intangible domains align with the valence of more concrete domains. One example is the conceptual metaphorical frame AFFECTION IS WARMTH which is rooted in the sense of touch/temperature, giving rise to numerous metaphorical expressions, such as “a warm hug” or “a cold-hearted person.” Some research indicates that social and physical warmth may indeed activate similar neural pathways associated with reward and pleasure (Inagaki & Eisenberger, 2013).^1^ Processing fluency, particularly motor fluency, serves as another key mechanism in forming valence associations (Reber et al., 1998; Winkielman & Cacioppo, 2001). An example is the association between motor fluency and the valence of horizontal spatial orientation, where right-side dominance in most individuals leads to a positive association with the right side. This bias is visible not only in language, as in the term “right-hand man,” but also in non-linguistic behaviors, with right-handers associating positively valenced concepts with rightward space and negatively valenced concepts with leftward space, a pattern that is reversed in left-handers (Casasanto, 2009). In turn, valence can also activate motor fluency simulation and bias perceptual judgment. For example, the presentation of positive words before a task requiring people to bisect vertical lines results in people bisecting lines biasing towards their dominant side (Milhau et al., 2017).

### Semantic mediation

Statistical learning from natural environments, magnitude matching, and valence matching often bypass the need for a deeper analysis of similarities in cross-domain mappings. However, in what way is time like money, jealousy like green, or thinking as a storm in the brain? We propose these mappings require mapping based on shared meanings and structures, a mechanism we termed as *semantic mediation*. In the sections that follow, we’ll explore three ways semantic mediation enables or moderate cross-domain mappings.

#### Semantic dimensions scaffold structural mappings

When faced with stimuli that lack overt similarities—a common challenge to cross-domain mappings—people can still align domains based on their common structure. At its core, structural mapping is about alignment on common dimensions across divergent sensory or conceptual domains. Relational language has been argued to be an important element in establishing some cross-domain mappings (Christie et al., 2007; Gentner, 2010; Gentner & Christie, 2010). Relational terms (words that describe relationships between things, like “over,” “under,” “between,” etc.) can facilitate the process of finding structural similarities between different domains. For example, children benefit from instructions that use these relational terms to guide alignment, aiding them in discerning the relational correspondences between two distinct domains (Christie & Gentner, 2014; L. Yuan et al., 2017).

Beyond facilitating explicit relational alignment, semantic dimensions also facilitate implicit structural mapping by aligning elements within a shared semantic space. The semantic coding hypothesis proposed by Martino and Marks (1999), holds that during cross-sensory mapping, experiences from different modalities are transformed from their sensory representations into more abstract, semantic codes. These codes are accessible to both our perceptual and conceptual systems allowing for semantic alignment across domains. In line with this hypothesis, linguistic cues, much like perceptual cues, are potent drivers of cross-sensory congruence. For example, words related to lightness, like *black* vs. *white* or semantically related words such as *day* vs. *night* can trigger congruence effects with high or low-pitched sounds in ways that parallel congruence effects involving pairs of nonlinguistic stimuli (Martino & Marks, 1999; using a similar paradigm shown in Fig. 3a. Word stimuli were used instead of a dot).

Semantic differentials, first introduced by Osgood in 1957, are a valuable tool for examining how semantic dimensions mediate cross-domain mappings. This method asks participants to rate stimuli based on scales anchored by polar adjectives, like “loud-quiet” or “small-large” (as shown in Fig. 1e–f). In pioneering work by Karwoski et al. (1942) demonstrated that even basic perceptual features such as visual brightness or auditory pitch, possess rich, domain-general conceptual connotations. Subsequent studies by L. Walker et al. (2012) attribute consistent cross-sensory mappings to the cross-activation between dimensions of shared connotative meaning. Similarly, when matching sensory stimuli like music with colors, participants chose colors that matched the music based on semantic meaning, such as whether the music and colors were complex or simple, lively or dreary (Palmer et al., 2013; Whiteford et al., 2018). Similarly, cross-conceptual mappings could be influenced by their conceptual alignments on shared abstract dimensions. For example, when asked “If a flute were a job, what job would it be?”, there was a surprising degree of consensus in people’s responses: 20% answered ‘teacher’, significantly above the baseline probability of 7% for listing ‘teacher’ as a type of job. These kinds of mappings between concepts from disparate semantic domains like *animal*, *job*, and *musical instruments* were best accounted for when using alignment on semantic dimensions such as speed, valence, and genderedness as predictors of similarity (Liu & Lupyan, 2023).

#### Lexical mediation

Languages abound with phrases like “loud colors,” “sweet sound,” and “high pitch” (Winter, 2016). This type of language is so ubiquitous that we often overlook its metaphorical roots. One possibility suggests that these expressions merely label pre-existing associations between different sensory domains and are not necessary for identifying the cross-domain similarity. For instance, the Kreung people of a remote tribe in northeastern Cambodia, whose language does not use spatial language for pitch, still associate pitch with elevation (Parkinson et al., 2012).

However, the view that metaphors simply reflect pre-existing associations is challenged by evidence that the strength of cross-domain mappings can be influenced by their frequency of use in a specific language (Casasanto et al., 2003; Fernandez-Prieto et al., 2017; Holler et al., 2022). For example, Dutch and English use “high” and “low” to describe pitch, while Farsi uses “thin” and “thick.” Although height-pitch and thickness-pitch correspondences are found in prelinguistic infants (Dolscheid et al., 2012; P. Walker et al., 2010), Dutch speakers incorporate irrelevant height information and ignore irrelevant thickness information when estimating pitch, whereas Farsi speakers incorporate irrelevant thickness information and ignore irrelevant height information (refer to Fig. 2a for a similar paradigm). Dutch speakers, after being trained to linguistically describe pitch as thick/thin, have demonstrated nonlinguistic thickness–pitch mappings similar to Farsi speakers (Dolscheid et al., 2013), suggesting language can play a causal role in shaping nonlinguistic mental representations of pitch. One explanation is that shared labels across domains may invite speakers to align sensory representations, drawing out similarities through structural alignment (Christie & Gentner, 2010; Gentner, 1983) in a way that gradually becomes consistent with their language.

In a similar vein, cross-conceptual mappings may benefit from the reuse of words. In the same way that we perceive and express the relationship between height and pitch or temperature and color, we may use similar metaphoric structures to understand abstract concepts. For example, we often employ spatial metaphors to describe power dynamics (“higher status”; “lower class”) and time (“looking forward to the future”; “leaving the past behind”). The habitual use of these metaphorical expressions could shape our conceptual frameworks, aligning them more closely with the linguistic patterns present in our language. For example, Swedish spatializes time in terms of length (long/short), while Spanish spatializes time in terms of amount (much/small-bit), a difference reflected in how much interference was created by spatial cues consisting of length vs. amount. Crucially, Swedish-Spanish bilinguals show varied interference effects depending on language context, indicating that the representation of time may be flexible and depend on the linguistic framework employed at the moment (Bylund & Athanasopoulos, 2017).

In addition to inviting speakers to align representations across domains in line with how they are coded in the language, lexical mediation may also influence the directionality of cross-domain mappings. For example, bidirectional mappings between time and space are observed in neonates (de Hevia et al., 2014; Lourenco & Longo, 2010) and some non-human primates (Merritt et al., 2010), suggesting an intuitive ability to associate duration with distance without the influence of language. However, in human adults, these mappings become asymmetric. For example, incongruent spatial information interferes with time estimation, but not vice versa (Casasanto & Boroditsky, 2008). This linguistic asymmetry is also evident in how metaphors describe time in terms of space (e.g., long/short time) more frequently than the reverse. The discrepancy in language use between human adults and neonates/non-human primates suggests that the asymmetry in linguistic metaphors could be a candidate causing this cognitive asymmetry. Nonetheless, the causal relationship between linguistic asymmetry and cognitive asymmetry in cross-domain mappings remains to be more rigorously investigated through empirical studies.

#### Linguistic markedness

Evidence for cross-domain mappings often involves correspondence between binary dimensions such as high/low, small/large, and good/bad. When individuals are explicitly asked to map between these binary options, they tend to generate parallel association for both poles. For instance, a higher pitch tends to be paired with brighter colors, whereas a lower pitch is paired with darker colors. However, when the automaticity of associations is tested, such as with speeded discrimination tasks, the nonparallel correspondence of dimensions emerges. For example, while people are faster and more accurate in recognizing high-pitched tones emitted from high spatial locations, low-pitched tones were not more quickly recognized when emitted from lower spatial locations (Bonetti & Costa, 2018). Likewise, there is a congruence effect for positive valence and higher spatial positions, but this effect is less pronounced or absent between negative valence and lower positions (Huang & Tse, 2015; Lakens, 2012; Lynott & Coventry, 2014). The nonparallel dimensional interaction is sought to be explained by semantic mediation via linguistic markedness.

Linguistic markedness refers to an asymmetrical relationship between elements where one element is considered more basic or default (unmarked) while the other is more specialized or derivative (marked). This concept applies across linguistic domains—for example, oral (unmarked) vs. nasal vowels in phonology, singular (unmarked) vs. plural nouns in morphology, and active (unmarked) vs. passive voice in syntax. Our focus here is on semantic or conceptual markedness, which applies to scalar adjectives and evaluative concepts. In pairs like tall/short, big/small, good/bad, and high/low, the first term is typically unmarked—serving as the default end of a dimension—while the second is marked, indicating contrast, limitation, or absence.

Everyday language reflects this asymmetry: asking how tall someone is does not imply that they are tall while asking how short someone is does imply shortness. Markedness is also used to explain why negation applies to one end but not the other, e.g., happy/unhappy, but sad/*unsad*.* It is suggested that the default, unmarked pole has a processing advantage over the marked pole (Clark, 1969; Clark & Brownell, 1975; Hommel et al., 2001; Seymour, 1974). When the unmarked pole of conceptual meaning (e.g., positive valence) and the unmarked pole of perceptual features (e.g., *high* vertical location) of a stimulus overlap, people’s processing is boosted. There’s no processing benefit otherwise, i.e., when the two marked poles overlap (e.g., negative valence and low vertical location), or having the unmarked pole corresponds to the marked pole (e.g., negative valence and high vertical location). To further test how linguistic markedness could be manipulated and affect cross-domain mappings, Lakens et al. (2012) increased the salience of negative words by making them more frequent, turning the negative end of the valence dimension into the default or unmarked pole. The results showed that increasing the frequency of negative words did indeed eliminate the congruence effect for positive words and high vertical locations.^2^

Appeals to linguistic markedness do not, however, explain cross-domain mappings involving metathetic dimensions that typically do not have linguistically marked and unmarked poles. For example, metathetic dimensions like sweet, sour, and bitter don’t fit into a marked/unmarked dichotomy and argument of linguistic markedness does not apply. In addition, linguistic markedness is not sufficient to elicit cross-domain congruence effects on its own. For example, when experiments used spatial schemas other than high/low (e.g., front/back, big/small), there was no congruence effect arising between space and pitch, despite the overlap of unmarked ends (Dolscheid & Casasanto, 2015).

To summarize, semantics can mediate cross-domain mappings in several ways. First, semantic dimensions can serve as a scaffold, providing structure to abstract alignment. Second, using the same words across domains (e.g., “long” for both time and distance) can help establish or reinforce these mappings. Lastly, the structural overlap in polarities could modify the automaticity of cross-domain mappings.

## Cross-domain mappings are both perceptual and conceptual

On hearing a melody, one might perceive its pitch, loudness, timbre, and tempo. The same melody may also evoke the sensation of a gentle breeze or the image of a serene moonlight—none of which are directly perceived. Similarly, when observing colors, attributes like saturation, hue, and brightness are immediately discernible. Yet colors also bear deeper conceptual associations; a particular shade of deep blue may conjure feelings and images associated with the expanse of the night sky. Are cross-domain mappings such as these experienced from aligning sensations like timbre and saturation, or are they conceived more in the abstract manner of associating melody and color similarly to the ambiance of a moonlit night?

The distinction between perception and conception is contentious. Some have argued that perception is “cognitively impenetrable”—and is strictly concerned with modal attributes: colors, shapes, smells, vibration. These perceptual inputs then feed into higher-level systems for further processing that is outside of perception proper (Fodor, 1983; Pylyshyn, 1999; Tye, 1995). Others (including some critics of cognitive penetrability) have argued that perceptual experience also encompasses higher-level content: seeing an object can include recognizing its category (Bayne, 2009), we can perceive causal relations (Kominsky & Scholl, 2020; Rolfs et al., 2013) as well as social and physical ones (Hafri & Firestone, 2021). We take the stance that there is a continuum from perceptual to higher-level conceptual processing with considerable interaction between lower-level and higher-level processes (Goldstone & Barsalou, 1998; Lupyan, 2015, 2017; Quinn & Eimas, 1997).

Some cross-domain mappings align more closely with the perceptual end of the continuum, and display rapid and automatic associations that are phenomenologically stubborn even when contradicted by explicit knowledge. A classic example is the McGurk effect, where an individual’s speech perception is markedly altered by seeing a speaker’s mouth make a different sound (e.g., seeing a mouth utter/ga/while hearing/ba/causes us to perceive a/da/; McGgurk & Macdonald, 1976). This effect shows how visual and auditory cues are rapidly integrated to form a coherent perceptual experience, often leading to a different interpretation than when auditory and visual cues are experienced in isolation. Similar phenomena include the ventriloquism effect, where visual cues influence the perceived location of sounds (Parise & Spence, 2008, 2009; usually explained by statistical learning), the interference of spatial information with numerical estimation (Dormal & Pesenti, 2007; usually explained by magnitude matching), and the alteration of taste experience by visual pleasantness (Ohla et al., 2012; usually explained by valence matching). These phenomena are not restricted to human adults; they have been observed in human infants and across animal species, suggesting the ability to perceptually integrate information from multiple sensory domains may have deep evolutionary roots. (For more evidence favoring different perceptual-level explanations, see Table 1 and Table 2.)

In contrast, some mappings are not directly derived from sensory input but are constructed through abstract reasoning. For example, metaphoric expressions like “Juliet is the sun” require abilities to draw meaningful parallels between two disparate domains that transcend perceptual correspondence (i.e., Juliet does not emit infrared radiation or have things orbiting around her). The partial projection between conceptual spaces is extensively discussed in Turner and Fauconnier’s theory of conceptual integration (Fauconnier & Turner, 1998, 2008). Another example is people use cross-domain mappings when trying to understand the model of an atom through an analogy between planetary systems and electrons orbiting around the nucleus, or when trying to understand an increase in crime through analogy to rampaging animals or a spreading virus (Thibodeau & Boroditsky, 2011). Unlike perceptual mechanisms, which support cross-domain mappings that are more consistent, stable, and universal, conceptual mechanisms support mappings that are more dynamic and variable, adapting to different frames in culture, language, and personal experience. As a result, people become more sensitive to cross-domain mappings encoded in their language (Dolscheid et al., 2013). While statistical learning, magnitude matching, and valence matching are primarily proposed to explain lower-level perceptual mechanisms,^3^ semantic mediation is primarily proposed to explain higher-level conceptual mechanisms. (For more evidence favoring conceptual-level explanations, see Table 1 and Table 2.)

Relatively lower-level mechanisms based on perceptual integration yield forms of cross-domain mappings similar to those observable in neonates or non-human animals, while higher-level mechanisms bring cross-domain mappings to their full-blown forms as observed in human adults. Through two case studies, we look into how cross-domain mappings we encounter in the real world are sculpted by both lower and higher-level mechanisms.

## Cross-sensory mappings are more conceptual than you think: the case of emotion-mediated mappings

One explanation for explaining the patterns of associations between stimuli like colors, shapes, music, and odors is emotion mediation (Arnheim, 1986; Levitan et al., 2014; Palmer et al., 2013; Whiteford et al., 2018). On this view, particular types of music and colors are associated to the extent that both evoke similar emotions. For example, happy music is aligned with happy colors (i.e., colors that evoke joy). But are such emotion-mediated mappings best thought of as being more on the perceptual or conceptual end?

Emotions have been argued to be perceivable entities by some researchers and conceptual constructs by others, representing two distinct theoretical traditions in understanding emotional processing. On the one hand, infants and animals—from rodents to primates—can differentiate emotional cues in facial/body expressions and vocalizations (e.g., Briefer, 2012; Grossmann, 2010). Cross-cultural studies have demonstrated that some basic emotions like happiness, sadness, anger, fear, disgust, and surprise are recognized with above-chance accuracy across diverse cultures (Ekman & Friesen, 1971; Elfenbein & Ambady, 2002). Such evidence has been used in support of emotions being rooted in some universal, perceivable components. On the other hand, it has also been shown that emotion recognition is partly mediated by language (Lindquist et al., 2006; Souter et al., 2021). People who speak different languages show some differences in emotion perception (Gendron et al., 2014). Such findings led researchers to argue for emotion categories being conceptual constructs rather than a “readout” of perceptual categories. To reconcile these two views, we can consider emotions as arising from varying activation levels along core affective dimensions like valence (an evaluative continuum) and arousal (a magnitude continuum; Barrett & Satpute, 2013; Lindquist et al., 2013). Meanwhile, language helps to abstract diverse physiological and behavioral patterns into more discrete emotion categories at higher conceptual levels. Discrete emotions, mediated by emotion concepts, likely involve semantic processing both when emotion categories are initially learned and when emotions are experienced and interpreted in real time (Jackson et al., 2019; Lindquist et al., 2006, 2015; Satpute & Lindquist, 2019, 2021).

Given that emotions function as both perceptual entities and conceptual constructs, we can understand cross-sensory mappings as operating at two levels. At a lower level, the correspondence between cross-domain sensory stimuli can be linked to attributes such as magnitude and valence in a relatively universal manner. At a higher level, the correspondence might be explained by alignments on semantic dimensions or linguistically encoded emotion categories. This hypothesis is supported by evidence showing that mappings between cross-sensory stimuli display both cultural universality and variability. Cross-culturally, consistent cross-sensory associations are observed when stimuli vary along core affective dimensions. For example, participants from both the U.S. and Mexico consistently matched fast-tempo, major-mode music to more saturated, lighter, and yellower colors. Colors and music strongly aligned on core dimensions like positive/negative and strong/weak (Palmer et al., 2013). When emotional content was statistically controlled for, the correlations between perceptual features (e.g., faster tempo to redder colors) of music and colors disappeared, with two latent affective factors—valence and arousal—accounting for most of the variance (Whiteford et al., 2018).

At the same time, participants from similar cultural and linguistic backgrounds tend to be more aligned in their associations between sensory stimuli and emotions. Research on music-emotion associations demonstrates that while certain basic emotions (e.g., happy/sad) are recognized across cultures, Western European participants (Germany and Norway) showed similar recognition patterns to each other, as did Asian participants (Korea and Indonesia) among themselves, with an in-group advantage for recognizing emotions from one’s own cultural background (Argstatter, 2016). In a study that included participants from 30 countries, linguistic and geographic proximity significantly predicted similarity in color-emotion associations, with linguistic distance being a stronger predictor than geographic distance (Jonauskaite et al., 2020). This interplay between perceptual mechanisms and conceptual mechanisms predicts both a strong universal consistency and local variations specific to people who speak the same language. Understanding which specific color-music associations vary across cultures and languages remain open questions.

## Cross-conceptual mappings are more perceptual than you think: The case of metaphors

Traditionally, metaphors have been understood as mappings between abstract and categorical conceptual representations. This means that metaphors are processed not by drawing direct parallels with literal, concrete features, but rather through more abstract associations. For instance, in the metaphor “time is a thief,” the focus is on the abstract qualities of time and theft (taking something away from us), rather than on a literal interpretation involving time wearing a mask or carrying a bag of stolen goods. This abstraction-focused approach to metaphor is supported by evidence suggesting that metaphor comprehension either requires inhibiting literal sensorimotor representations of the source (McGlone & Manfredi, 2001), or bypassing them entirely through direct access to abstract meanings (Glucksberg, 2008; Keysar, 1989).

However, where do the abstract qualities of a concept come from in the first place? Embodiment theory suggests that abstract metaphors are structured by our sensorimotor experiences (Ackerman et al., 2010; Boot & Pecher, 2010; Gibbs, 2006; Wilkowski et al., 2009; see also Desai, 2021; Khatin-Zadeh et al., 2023, for reviews). In conceptual metaphor theory—a major strand of embodied cognition, mappings usually run from richer, highly structured, experientially concrete domains (space, motion, force, bodily sensation) to sparser, abstract ones (time, emotion, social relations). For example, space is three-dimensional and directionless, while time is one-dimensional and directional. This asymmetry in structural properties allows time to “borrow” spatial structure in flexible ways, reflected in the linguistic patterns we use to talk—and think—about it. In English, time is often spatialized in two contrasting frames. In ego-moving expressions (e.g., “we’re coming up on Summer”), time is stationary and we move through it. In time-moving expressions (e.g., “Summer flew by”), time itself is in motion while we remain still. Bodily actions can bias temporal interpretation in line with these spatial frames. For instance, after pulling a chair toward themselves, participants are more likely to interpret “The meeting on Wednesday has been moved forward two days” as moving to Friday (ego-moving) rather than to Monday (time-moving; Boroditsky, 2000; Boroditsky & Ramscar, 2002). Cross-linguistic evidence further shows that languages vary in how they spatialize time—conceptualizing it as flowing from left to right, right to left, front to back, top to bottom, west to east, or even along the course of rivers. Consistent with their spatial frame of time, speakers of different languages mentally represent and reason about temporal sequences differently (Fuhrman & Boroditsky, 2010; R. E. Núñez & Sweetser, 2006; Santiago et al., 2007; Torralbo et al., 2006).

In support of the embodiment theory, behavioral studies show that performing, observing and even imagining compatible physical actions (such as grasping) facilitates the processing of conceptually related metaphors (such as “grasp the concept”; Gibbs, 2006; Gibbs et al., 2006; Horchak et al., 2014; Wilson & Gibbs, 2007). Conversely, simulating metaphors can also influence subsequent bodily judgments and behaviors (Gibbs, 2013; Perlman et al., 2014; Slepian & Ambady, 2014). For example, participants who heard about a successful relationship “moving along in a good direction” subsequently walked both longer in time and further in distance compared with those who heard about an unsuccessful relationship with the same metaphorical frame (Gibbs, 2013). At the neural level, it has been shown that processing texture and taste metaphors such “she had a rough day” (Lacey et al., 2012) and she looked at him sweetly (Citron & Goldberg, 2014) activates corresponding sensorimotor cortices. While such sensorimotor activations during metaphor comprehension could partly reflect associative spread from related literal senses and offer only correlational evidence, stronger causal evidence comes from studies using interference methods: for instance, disrupting motor cortex with transcranial magnetic stimulation (TMS) selectively impairs the comprehension of action-related metaphors (e.g., grasp an idea; Reilly et al., 2019; Willems et al., 2011), suggesting that sensorimotor systems might play a functional role in metaphor understanding (Casasanto, 2022).

The degree of sensorimotor involvement changes as metaphors become familiar. The structure-mapping theory (Gentner, 1983) and career-of-metaphor theory (Bowdle & Gentner, 2005) provide a possible mechanism for how repeated use of metaphoric sense leads to abstraction: Novel metaphors require active analogical mapping between source and target domains, often recruiting sensorimotor simulations of the source. Over time, repeated metaphorical uses crystallize into abstracted senses that can be accessed directly, without reactivating the literal, embodied features of the source domain. Supporting this mechanism, it was shown that novel metaphors more strongly activate sensorimotor features compared with familiar ones (Al-Azary & Katz, 2021; Desai et al., 2011). For example, when comprehending nominal metaphors (e.g., my lawyer is a shark), participants respond more quickly to literal, perceptual features of the source domain (e.g., *bite*) when the metaphor is novel, but respond more quickly to abstract, conventionalized features (e.g., *killer*) when the metaphor is familiar (Al-Azary & Katz, 2021).

Based on this embodied view of metaphor, cross-conceptual mappings, like cross-sensory mappings, are grounded in perceptual experiences. Concepts often have an embodied basis that is somewhat consistent across cultures. This is evident in widespread conceptual mappings such as those between time and space, good and up, or affection and warmth, which can be explained by experiential correlations in the environment, magnitude matching, or valence matching. Based on this perceptual foundation, cultures, and languages play a significant role in mediating and elaborating these mappings, leading to constraint variations in how relatively abstract concepts are understood in different cultures. While frequently used abstractions may develop more independent representations over time, their perceptual basis continues to provide a foundation for understanding, especially when learning novel abstract concepts.

## Future directions

The empirical findings reviewed in this article show that two previously remote phenomena: cross-sensory mapping and cross-conceptual mapping, are perhaps two manifestations of a global mechanism. Cross-domain mappings, in general, rely on a process that requires operation on both the level of perception and conception.

### What insights can we draw from studying cross-sensory mapping and cross-conceptual mapping together?

We have discussed a number of similarities between cross-sensory mappings and cross-conceptual mappings. However, empirical research uniting these mappings remains scarce. Should the mechanisms underlying these mappings be homogeneous, we might expect a positive transfer from one type of mapping to another. For instance, if individuals can adeptly comprehend novel metaphors or show strong analogical reasoning abilities, would they also exhibit greater consistency in cross-sensory mappings compared to individuals who struggle with metaphor comprehension or solving analogies? Moreover, do people tend to rely on the same mechanisms for different types of cross-domain mappings? Could bias in making one kind of cross-domain mapping predict people’s bias in making another kind of cross-domain mapping? For example, in music-color mappings, some individuals might rely more on statistical cues (e.g., certain genres of music often co-occur with specific colors in the environment, like rock music being associated with darker attire, or classical music being associated with yellow or deep red due to warm concert hall lighting), while others might depend more on semantic associations (e.g., happy music paired with bright, cheerful colors; sad music paired with dark, muted colors; Liu et al., 2024). Similarly, in cross-conceptual mappings, such as associating jobs with animals, one might project a police officer onto a dog given that police officers often work with dogs whereas another might project a police officer onto a lion, relying more on shared semantic attributes like authority and power. When statistical and semantic biases lead to different mappings, would a person’s bias in one type of cross-domain mapping predicts their bias in another type of mapping? For example, would those who associate sad classical music with yellow (statistical bias) or blue (semantic bias) tend to map police officers to dogs or lions, respectively?

### Can people’s cross-domain mappings tell us about cognitive abilities such as abstraction, creativity and inductive reasoning?

Our ability to identify similarities across seemingly disparate domains is a cognitive skill that broadly manifests in various similarity-based tasks previously used to investigate general cognitive abilities such as abstraction, creativity, and inductive reasoning. Consider, for instance, the classic “odd-one-out” paradigm for inductive reasoning. Individuals must identify the item that does not belong to a given set based on shared characteristics or conceptual relationships. An example set is horse, cow, and milk, where either milk or horse could be the odd one out, depending on whether the relevant feature is category membership (mammal) or context (milk as a product of cows; Duan & Lupyan, 2023). Ad hoc categorization tasks require participants to group ostensibly unrelated things under a novel category. For example, they might be asked to create a category for “items that bring comfort,” individuals need to identify abstract similarities that transcend typical categorization schemes and group items like a blanket, tea, and a book. Similarly, director-matcher tasks like *Codenames* (a popular word-guessing game) also relies on players’ ability to identify connections between words (e.g., Rissman et al., 2023), where players give one-word clues to help their teammates identify specific words from a grid, such as using the clue “water” to link the words “ocean,” “fish,” and “tears.” These tasks all involve drawing connections between seemingly disparate domains and probing our conceptual space in flexible and creative ways.

Preliminary evidence suggests that proficiency in cross-sensory and cross-conceptual mapping may be part of a domain-general creative ability. For instance, synesthesia—disproportionately observed among artists and other highly creative individuals (Dailey et al., 1997; Domino, 1989; Root-Bernstein & Root-Bernstein, 1999)—is associated with not only more frequent use of cross-sensory language but also more creative use of metaphors (Turner & Littlemore, 2023). This raises intriguing questions about whether creative skills in cross-mapping might transfer to other cognitive tasks. For example, might people who excel at cross-domain mapping also demonstrate greater creativity and flexibility in tasks like ad hoc categorization? do people who favor certain strategies or biases in mapping (e.g., relying on statistical co-occurrences) exhibit the same biases in tasks like odd-one-out*?* Are individuals who align closely in their cross-domain associations (e.g., both associating Bach with blue or nurses with violins) also more effective at communicating about clues in a word guessing game? Could there be a causal relationship whereby training people to do novel cross-domain mapping (e.g., “How is a tree like a family?”) enhances their domain-general abstract reasoning and cognitive flexibility, such as improving performance in tasks requiring ad hoc categorization, transferring solutions across context to solve new problems, and enhancing communication in collaborative settings?

### Does language play a causal role in cross-domain mappings?

Throughout this review, we have seen tantalizing hints of linguistic influences on cross-domain mappings, but its causal role remains murky. Does language simply reflect pre-existing mappings or does it actively shape how we connect disparate domains of experience?

Some cross-domain mappings are more automatic than others. For example, mappings between space and time are symmetrically automatic in early life (e.g., Srinivasan & Carey, 2010; see Table 1 and Table 2 for more examples) but become more asymmetrical in adulthood with inconsistent spatial information interfere with time estimation but not the reverse (Casasanto & Boroditsky, 2008; Winter et al., 2015), paralleled by the asymmetry in linguistic metaphors of using space to describe time than the reverse. However, to what extent does language drive this asymmetry? Longitudinal studies could track the development of specific cross-domain mappings from infancy through childhood, examining the temporal precedence of acquiring spatial metaphors for time in relation to changes in mapping symmetry. One could also manipulate the frequency of cross-domain linguistic mappings or teach novel cross-domain expressions, then test for changes in the automaticity of nonlinguistic mappings.

In addition, semantic mediation—one of the main mechanisms for cross-domain mapping—is usually operationalized by measuring alignment on semantic dimensions that are defined by linguistic anchors. But what is the relationship between language and the semantic dimensions that align cross-domain constructs? One possibility is that semantic space is reducible to basic constructs like valence and magnitude, with language merely reflecting these universal dimensions. An alternative possibility is that language not only reflects the semantic space but also programs it. For example, emotion literature has shown that the development of language supports the growth of emotion concept representations from a simple “positive or negative” dichotomy in childhood to a more multidimensional structure in adulthood (Nook et al., 2017). Emotion concepts as attached to emotion words—such as “surprise,” “grateful,” “envy,” “nostalgia,” and “anxiety”—help us not only articulate but also perceive discrete emotions beyond a simple positive-negative scale (Gendron et al., 2012; Lindquist et al., 2014, 2015; Lindquist & Gendron, 2013). Similarly, language goes beyond simply labeling quantities as “more” or “less” but refines our understanding of magnitude by providing us with the vocabulary needed to express, operate over, and memorize (Frank et al., 2008), and represent (Frank et al., 2012; Pitt et al., 2022) large and abstract magnitude (e.g., social status) with finer precision and detail. However, the causal role of language for semantic alignment between domains remains an empirical question. One can examine if people speaking different languages perform differently on cross-domain mappings that require semantic mediation. For example, color semantics might be different across languages, then people might associate the same music with different colors. This would be correlational though—but more causal role could be supported by experimentally manipulating language and examine the change in cross-domain mapping, such as asking multilingual people to do the same task with different linguistic contexts, or teach people to use novel linguistic descriptors for previously unconnected cross-domain stimuli and see if people prefer different mappings when their linguistic system is moderated. To further disentangle the effects of long-term linguistic experience in shaping semantics from the immediate, online influence of language on probing semantics, one can use verbal interference tasks to disrupt linguistic processing and observing effects on the pattern of cross-domain judgments. For example, are people speaking different languages behaving more/less alike when they cannot use language for semantically mediated cross-domain mappings? Are people more or less consistent when they cannot use language?

Another question arises from observations that individuals can perform cross-domain mappings even in the absence of direct sensory experience. For example, despite their inability to visually perceive color, congenitally blind individuals are able to map colors onto semantic dimensions in a manner similar to sighted individuals (Lenci et al., 2013; Saysani, 2019; Saysani et al., 2018, 2021; Shepard & Cooper, 1992). This capability is presumably due to their ability to acquire knowledge and deduce the conceptual structure of color space from language (J. S. Kim et al., 2021; Lupyan et al., 2020; Lupyan & Winter, 2018; Saysani et al., 2018, 2021; van Paridon et al., 2021). Research could further explore how language enables cross-domain mappings, especially in cases where individuals lack the appropriate sensory inputs for learning relevant domain knowledge perceptually. This exploration might involve studying populations with varying perceptual impairments to understand how they achieve conceptual mappings in the absence of typical sensory experiences. Similarly, examining individuals with language impairments could reveal insights into how deficits in linguistic capabilities affect the ability to form cross-domain mappings.

Finally, the remarkable cross-domain mapping abilities of large language models (LLMs) offer a new frontier for exploration (Motoki et al., 2024; Yehudai et al., 2024). Language models (e.g., Devlin, 2018; OpenAI, 2023; Radford et al., 2019) trained solely on text, can generate sensible mappings between disparate domains. This hints at the wealth of cross-domain information encoded in language itself. By comparing LLMs trained on different language corpora to human performance across cultures, we might identify which mappings are learnable from language alone and which require direct sensory experience.

## Conclusion

We sought to bring together two important areas of cognitive science that have traditionally been studied separately: cross-sensory mappings and cross-conceptual mappings. By examining these phenomena side by side, we argue that they are better understood as two manifestations of common underlying mechanisms. Specifically, we identified four key mechanisms underlying both types of cross-domain mappings: statistical learning from environmental regularities, matching based on magnitude, valence, and mediation through semantic associations.

While statistical learning, magnitude matching, and valence matching, arise early in development and are shared with other species, semantic mediation involves higher-order process such as abstract reasoning and symbolic interpretation. Crucially, we argue that what may appear as purely perceptual associations involve conceptual mediation, while seemingly abstract conceptual mappings are grounded in perceptual experiences. The interplay between perceptual and conceptual mechanisms is exemplified in phenomena like emotion-mediated mappings, where basic dimensions like valence and arousal interact with linguistically-mediated emotion concepts to produce rich and nuanced associations between disparate sensory domains. Similarly, conceptual metaphors exemplify how abstract concepts are often scaffolded upon and shaped by embodied, perceptual experiences.

Looking ahead, we propose integrating research on cross-sensory and cross-conceptual mappings to empirically examine shared cognitive mechanisms and explore how cross-domain mappings may inform our understanding of broader cognitive skills such as abstraction and creativity. Crucially, we advocate for rigorous investigation into the mechanisms through which language might causally influence cross-domain mappings, which will contribute to the broader discussion of how linguistic experience not only reflects mental representations but potentially plays an active role in shaping and programming them.

## Data Availability

All data and materials reviewed in this paper are from published sources cited in the references.

## Footnotes

1 However, the nonlinguistic evidence on temperature’s effects on prosocial behavior is mixed. Williams and Bargh (2008a) reported increased prosocial behavior with physical warmth, but subsequent replications, like those by Chabris et al. (2019), have inconsistent results. A recent meta-analysis by Lynott et al. (2023) suggests the effect may be unreliable, highlighting the need for more research with clear definitions and ecologically valid measures to clarify the relationship between temperature experience and social behavior.

2 However, the manipulation did not entirely reverse the congruence effect: even though the negative pole became the default end of valence, the study did not find a new congruence effect for negative words and high vertical locations.

3 Magnitude matching, valence matching accounts, and statistical learning from the natural environment can sometimes be challenging to differentiate. Indeed, association between *affection is warmth* could be jointly explained by statistical learning and valence matching given the fact that infants experience affection and warmth together and they are both pleasant experiences. Similarly, explaining the mapping between GOOD and DOMINANT can be explained by appealing both to magnitude matching (people associate more intense emotions with the right and less intense emotions with the left regardless of valence, which aligns with how other magnitudes (like numbers) are mentally mapped onto a left-to-right mental line; Holmes & Lourenco, 2011) and valence matching (people associate positive emotions with their dominant side of space and negative emotions with their non-dominant side; Pitt & Casasanto, 2018). Seen through the lens of optimal cue integration (e.g., Ernst, 2006; see also Spence, 2011, for a review), data from one domain can inform prior expectations of other domains, influencing our perception and interpretation of subsequent information. The congruent cross-sensory attributes, such as associating a food’s color with its taste, the magnitude of sound with the magnitude of physical size, the pleasant smell with a pleasant taste, can all be viewed as coupling priors guiding the brain’s integration of cross-domain information, while incongruent attributes hinder it.

## Declarations

### Open practices statement

This article is a review and does not report original data. All information sources used in this review are cited in the reference list. No experiments were preregistered for this review.

## References

[CR1] Ackerman, J. M., Nocera, C. C., & Bargh, J. A. (2010). Incidental haptic sensations influence social judgments and decisions. *Science,**328*(5986), 1712–1715. 10.1126/science.1189993 20576894 10.1126/science.1189993 PMC3005631

[CR2] Adachi, I. (2014). Spontaneous spatial mapping of learned sequence in chimpanzees: Evidence for a SNARC-like effect. *PLoS One,**9*(3), Article e90373. 10.1371/journal.pone.0090373 24643044 10.1371/journal.pone.0090373 PMC3958337

[CR3] Akmajian, A., Demer, R. A., Farmer, A. K., & Harnish, R. M. (2001). *Linguistics: An introduction to language and communication*. MIT Press.

[CR4] Al-Azary, H., & Katz, A. N. (2021). Do metaphorical sharks bite? Simulation and abstraction in metaphor processing. *Memory & Cognition,**49*(3), 557–570. 10.3758/s13421-020-01109-2 33140133 10.3758/s13421-020-01109-2

[CR5] Anelli, F., Peters-Founshtein, G., Shreibman, Y., Moreh, E., Forlani, C., Frassinetti, F., & Arzy, S. (2018). Nature and nurture effects on the spatiality of the mental time line. *Scientific Reports*. 10.1038/s41598-018-29584-3 30076378 10.1038/s41598-018-29584-3 PMC6076263

[CR6] Anikin, A., & Johansson, N. (2019). Implicit associations between individual properties of color and sound. *Attention, Perception, & Psychophysics,**81*(3), 764–777. 10.3758/s13414-018-01639-7 10.3758/s13414-018-01639-7 PMC6407832 30547381

[CR7] Antović, M., Mitić, J., & Benecasa, N. (2020). Conceptual rather than perceptual: Cross-modal binding of pitch sequencing is based on an underlying schematic structure. *Psychology of Music,**48*(1), 84–104. 10.1177/0305735618785242

[CR8] Argstatter, H. (2016). Perception of basic emotions in music: Culture-specific or multicultural? *Psychology of Music,**44*(4), 674–690. 10.1177/0305735615589214

[CR9] Arnheim, R. (1986). New essays on the psychology of art. *University of California Press*. 10.1525/9780520907843

[CR10] Athanasopoulos, P., & Bylund, E. (2023). Cognitive restructuring: Psychophysical measurement of time perception in bilinguals. *Bilingualism, Language and Cognition*. 10.1017/S1366728922000876

[CR11] Barrett, L. F., & Satpute, A. B. (2013). Large-scale brain networks in affective and social neuroscience: Towards an integrative functional architecture of the brain. *Current Opinion in Neurobiology,**23*(3), 361–372. 10.1016/j.conb.2012.12.012 23352202 10.1016/j.conb.2012.12.012 PMC4119963

[CR12] Bayne, T. (2009). Perception and the reach of phenomenal content. *The Philosophical Quarterly,**59*(236), 385–404. 10.1111/j.1467-9213.2009.631.x

[CR13] Belin, P., McAdams, S., Smith, B., Savel, S., Thivard, L., Samson, S., & Samson, Y. (1998). The functional anatomy of sound intensity discrimination. *The Journal of Neuroscience,**18*(16), 6388–6394. 10.1523/JNEUROSCI.18-16-06388.1998 9698330 10.1523/JNEUROSCI.18-16-06388.1998 PMC6793181

[CR14] Bender, A., & Beller, S. (2014). Mapping spatial frames of reference onto time: A review of theoretical accounts and empirical findings. *Cognition,**132*(3), 342–382. 10.1016/j.cognition.2014.03.016 24873738 10.1016/j.cognition.2014.03.016

[CR15] Berridge, K. C., & Kringelbach, M. L. (2015). Pleasure systems in the brain. *Neuron,**86*(3), 646–664. 10.1016/j.neuron.2015.02.018 25950633 10.1016/j.neuron.2015.02.018 PMC4425246

[CR16] Bestelmeyer, P. E. G., Kotz, S. A., & Belin, P. (2017). Effects of emotional valence and arousal on the voice perception network. *Social Cognitive and Affective Neuroscience,**12*(8), 1351–1358. 10.1093/scan/nsx059 28449127 10.1093/scan/nsx059 PMC5597854

[CR17] Blazhenkova, O., & Kumar, M. M. (2018). Angular versus curved shapes: Correspondences and emotional processing. *Perception,**47*(1), 67–89. 10.1177/0301006617731048 28927319 10.1177/0301006617731048

[CR18] Blumberg, M. S., Efimova, I. V., & Alberts, J. R. (1992). Ultrasonic vocalizations by rat pups: The primary importance of ambient temperature and the thermal significance of contact comfort. *Developmental Psychobiology,**25*(4), 229–250. 10.1002/dev.420250402 1624055 10.1002/dev.420250402

[CR19] Bonetti, L., & Costa, M. (2018). Pitch-verticality and pitch-size cross-modal interactions. *Psychology of Music,**46*(3), 340–356. 10.1177/0305735617710734

[CR20] Boot, I., & Pecher, D. (2010). Similarity is closeness: Metaphorical mapping in a conceptual task. *Quarterly Journal of Experimental Psychology,**63*(5), 942–954. 10.1080/17470210903134351 10.1080/17470210903134351 19731189

[CR21] Boroditsky, L. (2000). Metaphoric structuring: Understanding time through spatial metaphors. *Cognition,**75*(1), 1–28. 10815775 10.1016/s0010-0277(99)00073-6

[CR22] Boroditsky, L. (2008). Do English and Mandarin speakers think differently about time? *Proceedings of the Annual Meeting of the Cognitive Science Society*, *30*(30).

[CR23] Boroditsky, L., Fuhrman, O., & McCormick, K. (2011). Do English and Mandarin speakers think about time differently? *Cognition,**118*(1), 123–129. 21030013 10.1016/j.cognition.2010.09.010

[CR24] Boroditsky, L., & Gaby, A. (2010). Remembrances of times East: Absolute spatial representations of time in an Australian Aboriginal community. *Psychological Science,**21*(11), 1635–1639. 20959511 10.1177/0956797610386621

[CR25] Boroditsky, L., & Ramscar, M. (2002). The roles of body and mind in abstract thought. *Psychological Science,**13*(2), 185–189. 11934006 10.1111/1467-9280.00434

[CR26] Bottini, R., & Casasanto, D. (2013). Space and time in the child’s mind: Metaphoric or ATOMic? *Frontiers in Psychology*, *4*. https://www.frontiersin.org/article/10.3389/fpsyg.2013.00803 10.3389/fpsyg.2013.00803 PMC3817359 24204352

[CR27] Boulenger, V., Hauk, O., & Pulvermüller, F. (2009). Grasping ideas with the motor system: Semantic somatotopy in idiom comprehension. *Cerebral Cortex,**19*(8), 1905–1914. 10.1093/cercor/bhn217 19068489 10.1093/cercor/bhn217 PMC2705699

[CR28] Bowdle, B. F., & Gentner, D. (2005). The career of metaphor. *Psychological Review,**112*(1), 193. 15631593 10.1037/0033-295X.112.1.193

[CR29] Bradley, M., & Lang, P. (1999). *Affective Norms for English Words (ANEW): Instruction manual and affective ratings*. https://www.semanticscholar.org/paper/Affective-Norms-for-English-Words-(ANEW)%3A-Manual-Bradley-Lang/c765eb0a31849361d829b24e173a37bab0919892

[CR30] Briefer, E. F. (2012). Vocal expression of emotions in mammals: Mechanisms of production and evidence. *Journal of Zoology,**288*(1), 1–20. 10.1111/j.1469-7998.2012.00920.x

[CR31] Brinton, L. J. (2000). *The structure of modern English: A linguistic introduction*. Johns Benjamins. https://books.google.com/books?hl=en&lr=&id=7Zyz0A6bXWEC&oi=fnd&pg=PR13&dq=The+Structure+of+Modern+English&ots=sATmGQfZ4h&sig=DKFp0_sxNMuUKxinwRB-F9aX_VY

[CR32] Bruzzi, E., Talamini, F., Priftis, K., & Grassi, M. (2017). A smarc effect for loudness. *I-Perception,**8*(6), Article 2041669517742175. 10.1177/2041669517742175 29201342 10.1177/2041669517742175 PMC5700794

[CR33] Bueti, D., & Walsh, V. (2009). The parietal cortex and the representation of time, space, number and other magnitudes. *Philosophical Transactions of the Royal Society B: Biological Sciences,**364*, 1831–1840. 10.1098/rstb.2009.0028 10.1098/rstb.2009.0028 PMC2685826 19487186

[CR34] Bulf, H., de Hevia, M. D., & Macchi Cassia, V. (2016). Small on the left, large on the right: Numbers orient visual attention onto space in preverbal infants. *Developmental Science,**19*(3), 394–401. 10.1111/desc.12315 26074348 10.1111/desc.12315

[CR35] Bulusu, V., & Lazar, L. (2024). Crossmodal associations between naturally occurring tactile and sound textures. *Perception,**53*(4), 219–239. 10.1177/03010066231224557 38304994 10.1177/03010066231224557

[CR36] Bylund, E., & Athanasopoulos, P. (2017). The whorfian time warp: Representing duration through the language hourglass. *Journal of Experimental Psychology, General,**146*(7), 911–916. 10.1037/xge0000314 28447839 10.1037/xge0000314

[CR37] Cardillo, E. R., McQuire, M., & Chatterjee, A. (2018). Selective metaphor impairments after left, not right, hemisphere injury. *Frontiers in Psychology*. 10.3389/fpsyg.2018.02308 30559690 10.3389/fpsyg.2018.02308 PMC6286990

[CR38] Casasanto, D. (2009). Embodiment of abstract concepts: Good and bad in right- and left-handers. *Journal of Experimental Psychology: General,**138*(3), 351–367. 10.1037/a0015854 19653795 10.1037/a0015854

[CR39] Casasanto, D. (2022). *Embodied semantics.* In F. T. Li (Ed.), *Handbook of cognitive semantics.* Brill*.* http://www.casasanto.com/papers/Casasanto%20Embodied%C2%A0Semantics%C2%A02022.pdf

[CR40] Casasanto, D., & Boroditsky, L. (2008). Time in the mind: Using space to think about time. *Cognition,**106*(2), 579–593. 17509553 10.1016/j.cognition.2007.03.004

[CR41] Casasanto, D., & Chrysikou, E. G. (2011). When left is “right”: Motor fluency shapes abstract concepts. *Psychological Science,**22*(4), 419–422. 10.1177/0956797611401755 21389336 10.1177/0956797611401755

[CR42] Casasanto, D., Phillips, W., & Boroditsky, L. (2003). Do we think about music in terms of space? Metaphoric representation of musical pitch. *Proceedings of the Annual Meeting of the Cognitive Science Society*, *25*(25).

[CR43] Casasola, M. (2005). Can language do the driving? The effect of linguistic input on infants’ categorization of support spatial relations. *Developmental Psychology,**41*(1), 183–192. 10.1037/0012-1649.41.1.183 15656748 10.1037/0012-1649.41.1.183 PMC2696172

[CR44] Chabris, C. F., Heck, P. R., Mandart, J., Benjamin, D. J., & Simons, D. J. (2019). No evidence that experiencing physical warmth promotes interpersonal warmth. *Social Psychology,**50*(2), 127–132. 10.1027/1864-9335/a000361

[CR45] Chiou, R., & Rich, A. N. (2012). Cross-modality correspondence between pitch and spatial location modulates attentional orienting. *Perception,**41*(3), 339–353. 10.1068/p7161 22808586 10.1068/p7161

[CR46] Christie, S., & Gentner, D. (2010). Where hypotheses come from: Learning new relations by structural alignment. *Journal of Cognition and Development,**11*(3), 356–373. 10.1080/15248371003700015

[CR47] Christie, S., & Gentner, D. (2014). Language helps children succeed on a classic analogy task. *Cognitive Science,**38*(2), 383–397. 10.1111/cogs.12099 24215433 10.1111/cogs.12099

[CR48] Christie, S., Gentner, D., Vosniadou, S., & Kayser, D. (2007). Relational similarity in identity relation: The role of language. *Proceedings of the Second European Cognitive Science Conference*, 601–666.

[CR49] Chuquichambi, E. G., Munar, E., Spence, C., & Velasco, C. (2024). Individual differences in sensitivity to taste-shape crossmodal correspondences. *Food Quality and Preference,**115*, Article 105110. 10.1016/j.foodqual.2024.105110

[CR50] Citron, F. M. M., & Goldberg, A. E. (2014). Metaphorical sentences are more emotionally engaging than their literal counterparts. *Journal of Cognitive Neuroscience,**26*(11), 2585–2595. 10.1162/jocn_a_00654 24800628 10.1162/jocn_a_00654

[CR51] Clark, H. H. (1969). Linguistic processes in deductive reasoning. *Psychological Review,**76*(4), 387–404. 10.1037/h0027578

[CR52] Clark, H. H., & Brownell, H. H. (1975). Judging up and down. *Journal of Experimental Psychology. Human Perception and Performance,**1*(4), 339–352. 10.1037/0096-1523.1.4.339 1185121 10.1037//0096-1523.1.4.339

[CR53] Cohen Kadosh, R., Cohen Kadosh, K., & Henik, A. (2008). When brightness counts: The neuronal correlate of numerical-luminance interference. *Cerebral Cortex (New York, N.Y. : 1991),**18*(2), 337–343. 10.1093/cercor/bhm058 17556772 10.1093/cercor/bhm058

[CR54] Cohen Kadosh, R., & Walsh, V. (2009). Numerical representation in the parietal lobes: Abstract or not abstract? *Behavioral and Brain Sciences,**32*(3–4), 313–328. 10.1017/S0140525X09990938 19712504 10.1017/S0140525X09990938

[CR55] Crawford, L. E., Margolies, S. M., Drake, J. T., & Murphy, M. E. (2006). Affect biases memory of location: Evidence for the spatial representation of affect. *Cognition and Emotion,**20*(8), 1153–1169. 10.1080/02699930500347794

[CR56] Crisinel, A.-S., & Spence, C. (2009). Implicit association between basic tastes and pitch. *Neuroscience Letters,**464*(1), 39–42. 10.1016/j.neulet.2009.08.016 19679162 10.1016/j.neulet.2009.08.016

[CR57] Crisinel, A.-S., & Spence, C. (2010). A sweet sound? Food names reveal implicit associations between taste and pitch. *Perception,**39*(3), 417–425. 10.1068/p6574 20465176 10.1068/p6574

[CR58] Crisinel, A.-S., & Spence, C. (2010). As bitter as a trombone: Synesthetic correspondences in nonsynesthetes between tastes/flavors and musical notes. *Attention, Perception & Psychophysics,**72*(7), 1994–2002. 10.3758/APP.72.7.1994 20952795

[CR59] Crisinel, A.-S., & Spence, C. (2011). Crossmodal associations between flavoured milk solutions and musical notes. *Acta Psychologica,**138*(1), 155–161. 10.1016/j.actpsy.2011.05.018 21696696 10.1016/j.actpsy.2011.05.018

[CR60] Crisinel, A.-S., & Spence, C. (2012). A fruity note: Crossmodal associations between odors and musical notes. *Chemical Senses,**37*(2), 151–158. 10.1093/chemse/bjr085 21852708 10.1093/chemse/bjr085

[CR61] Cuturi, L. F., Cappagli, G., Tonelli, A., Cocchi, E., & Gori, M. (2021). Perceiving size through sound in sighted and visually impaired children. *Cognitive Development,**60*, Article 101125. 10.1016/j.cogdev.2021.101125

[CR62] Dahl, C. D., & Adachi, I. (2013). Conceptual metaphorical mapping in chimpanzees (*Pan troglodytes*). *eLife,**2*, Article e00932. 10.7554/eLife.00932 24151544 10.7554/eLife.00932 PMC3798977

[CR63] Dailey, A., Martindale, C., & Borkum, J. (1997). Creativity, synesthesia, and physiognomic perception. *Creativity Research Journal,**10*(1), 1–8. 10.1207/s15326934crj1001_1

[CR64] de Hevia, M. D., Izard, V., Coubart, A., Spelke, E. S., & Streri, A. (2014). Representations of space, time, and number in neonates. *Proceedings of the National Academy of Sciences of the United States of America,**111*(13), 4809–4813. 10.1073/pnas.1323628111 24639511 10.1073/pnas.1323628111 PMC3977279

[CR65] de Hevia, M. D., & Spelke, E. S. (2010). Number-space mapping in human infants. *Psychological Science,**21*(5), 653–660. 10.1177/0956797610366091 20483843 10.1177/0956797610366091 PMC3129621

[CR66] de la Vega, I., Dudschig, C., De Filippis, M., Lachmair, M., & Kaup, B. (2013). Keep your hands crossed: The valence-by-left/right interaction is related to hand, not side, in an incongruent hand–response key assignment. *Acta Psychologica,**142*(2), 273–277. 10.1016/j.actpsy.2012.12.011 23376138 10.1016/j.actpsy.2012.12.011

[CR67] de Valk, J. M., Wnuk, E., Huisman, J. L. A., & Majid, A. (2017). Odor–color associations differ with verbal descriptors for odors: A comparison of three linguistically diverse groups. *Psychonomic Bulletin & Review,**24*(4), 1171–1179. 10.3758/s13423-016-1179-2 27783225 10.3758/s13423-016-1179-2 PMC5570805

[CR68] Dehaene, S., Bossini, S., & Giraux, P. (1993). The mental representation of parity and number magnitude. *Journal of Experimental Psychology: General,**122*(3), 371–396. 10.1037/0096-3445.122.3.371

[CR69] Delgado, J., Pereira, R., Ferreira, M. F., Farinha-Fernandes, A., Guerreiro, J. C., Faustino, B., & Ventura, P. (2020). Sound symbolism is modulated by linguistic experience. *Revista da Associacao Portuguesa de Linguistica,**7*, 137–150.

[CR70] Demattè, M. L., Sanabria, D., & Spence, C. (2006). Cross-modal associations between odors and colors. *Chemical Senses,**31*(6), 531–538. 10.1093/chemse/bjj057 16648449 10.1093/chemse/bjj057

[CR71] Demattè, M. L., Sanabria, D., & Spence, C. (2009). Olfactory discrimination: When vision matters? *Chemical Senses,**34*(2), 103–109. 10.1093/chemse/bjn055 18794200 10.1093/chemse/bjn055

[CR72] Deroy, O., Crisinel, A.-S., & Spence, C. (2013). Crossmodal correspondences between odors and contingent features: Odors, musical notes, and geometrical shapes. *Psychonomic Bulletin & Review,**20*(5), 878–896. 10.3758/s13423-013-0397-0 23463615 10.3758/s13423-013-0397-0

[CR73] Desai, R. H. (2021). Are metaphors embodied? The neural evidence. *Psychological Research*. 10.1007/s00426-021-01604-4 10.1007/s00426-021-01604-4 PMC10171917 34762153

[CR74] Desai, R. H., Binder, J. R., Conant, L. L., Mano, Q. R., & Seidenberg, M. S. (2011). The neural career of sensory-motor metaphors. *Journal of Cognitive Neuroscience,**23*(9), 2376–2386. 21126156 10.1162/jocn.2010.21596 PMC3131459

[CR75] Desai, R. H., Conant, L. L., Binder, J. R., Park, H., & Seidenberg, M. S. (2013). A piece of the action: Modulation of sensory-motor regions by action idioms and metaphors. *NeuroImage,**83*, 862–869. 10.1016/j.neuroimage.2013.07.044 23891645 10.1016/j.neuroimage.2013.07.044 PMC3819432

[CR76] Devlin, J. (2018). Bert: Pre-training of deep bidirectional transformers for language understanding. *arXiv Preprint.* arXiv:1810.04805.

[CR77] Dingemanse, M., Blasi, D. E., Lupyan, G., Christiansen, M. H., & Monaghan, P. (2015). Arbitrariness, iconicity, and systematicity in language. *Trends in Cognitive Sciences,**19*(10), 603–615. 10.1016/j.tics.2015.07.013 26412098 10.1016/j.tics.2015.07.013

[CR78] Dolscheid, S., & Casasanto, D. (2015). Spatial congruity effects reveal metaphorical thinking, not polarity correspondence. *Frontiers in Psychology*, *6*. https://www.frontiersin.org/article/10.3389/fpsyg.2015.01836 10.3389/fpsyg.2015.01836 PMC4659873 26635713

[CR79] Dolscheid, S., Çelik, S., Erkan, H., Küntay, A., & Majid, A. (2020). Space–pitch associations differ in their susceptibility to language. *Cognition,**196*, Article 104073. 10.1016/j.cognition.2019.104073 31810048 10.1016/j.cognition.2019.104073

[CR80] Dolscheid, S., Çelik, S., Erkan, H., Küntay, A., & Majid, A. (2023). Children’s associations between space and pitch are differentially shaped by language. *Developmental Science,**26*(5), Article e13341. 10.1111/desc.13341 36315982 10.1111/desc.13341

[CR81] Dolscheid, S., Hunnius, S., Casasanto, D., & Majid, A. (2012). *The Sound of Thickness: Prelinguistic Infants’ Associations of Space and Pitch*. 8. 10.1177/0956797614528521 24899170

[CR82] Dolscheid, S., Hunnius, S., Casasanto, D., & Majid, A. (2014). Prelinguistic infants are sensitive to space–pitch associations found across cultures. *Psychological Science,**25*(6), 1256–1261. 10.1177/0956797614528521 24899170 10.1177/0956797614528521

[CR83] Dolscheid, S., Shayan, S., Majid, A., & Casasanto, D. (2013). The thickness of musical pitch: Psychophysical evidence for linguistic relativity. *Psychological Science,**24*(5), 613–621. 10.1177/0956797612457374 23538914 10.1177/0956797612457374

[CR84] Domino, G. (1989). Synesthesia and creativity in fine arts students: An empirical look. *Creativity Research Journal,**2*(1/2), 17–29. 10.1080/10400418909534297

[CR85] Dormal, V., & Pesenti, M. (2007). Numerosity-length interference. *Experimental Psychology,**54*(4), 289–297. 10.1027/1618-3169.54.4.289 17953149 10.1027/1618-3169.54.4.289

[CR86] Drucker, C. B., & Brannon, E. M. (2014). Rhesus monkeys (*Macaca mulatta*) map number onto space. *Cognition,**132*(1), 57–67. 10.1016/j.cognition.2014.03.011 24762923 10.1016/j.cognition.2014.03.011 PMC4031030

[CR87] Duan, Y., & Lupyan, G. (2023). Divergence in word meanings and its consequence for communication. *Proceedings of the Annual Meeting of the Cognitive Science Society*, *45*(45). https://escholarship.org/uc/item/0dp4790t

[CR88] Eitan, Z., Schupak, A., Gotler, A., & Marks, L. E. (2014). Lower pitch is larger, yet falling pitches shrink: Interaction of pitch change and size change in speeded discrimination. *Experimental Psychology,**61*(4), 273–284. 10.1027/1618-3169/a000246 24351984 10.1027/1618-3169/a000246

[CR89] Ekman, P., & Friesen, W. V. (1971). Constants across cultures in the face and emotion. *Journal of Personality and Social Psychology,**17*(2), 124–129. 10.1037/h0030377 5542557 10.1037/h0030377

[CR90] Elfenbein, H. A., & Ambady, N. (2002). On the universality and cultural specificity of emotion recognition: A meta-analysis. *Psychological Bulletin,**128*(2), 203–235. 10.1037/0033-2909.128.2.203 11931516 10.1037/0033-2909.128.2.203

[CR91] Emmorey, K. (2014). Iconicity as structure mapping. *Philosophical Transactions of the Royal Society B: Biological Sciences,**369*(1651), Article 20130301. 10.1098/rstb.2013.0301 10.1098/rstb.2013.0301 PMC4123680 25092669

[CR92] Ernst, M. O. (2006). A Bayesian view on multimodal integration cue. In G. Knoblich, I. M. Thornton, M. Grosjean, & M. Shiffrar (Eds.), *Human body perception from the inside out: Advances in visual cognition* (pp. 105–131). Oxford University Press.

[CR93] Evans, K. K., & Treisman, A. (2010). Natural cross-modal mappings between visual and auditory features. *Journal of Vision,**10*(1), Article 6. 10.1167/10.1.6 PMC2920420 20143899

[CR94] Fauconnier, G., & Turner, M. (1998). Conceptual integration networks. *Cognitive Science,**22*(2), 133–187. 10.1016/S0364-0213(99)80038-X

[CR95] Fauconnier, G., & Turner, M. (2008). Rethinking metaphor. In R. W. Gibbs Jr. (Ed.), *The Cambridge Handbook of Metaphor and Thought* (pp. 53–66). Cambridge University Press. 10.1017/CBO9780511816802.005

[CR96] Fernández-Prieto, I., Navarra, J., & Pons, F. (2015). How big is this sound? Crossmodal association between pitch and size in infants. *Infant Behavior and Development,**38*, 77–81. 10.1016/j.infbeh.2014.12.008 25617593 10.1016/j.infbeh.2014.12.008

[CR97] Fernandez-Prieto, I., Spence, C., Pons, F., & Navarra, J. (2017). Does language influence the vertical representation of auditory pitch and loudness? *I-Perception,**8*(3), Article 2041669517716183. 10.1177/2041669517716183 28694959 10.1177/2041669517716183 PMC5484432

[CR98] Firestone, C., & Scholl, B. J. (2014). “Top-down” effects where none should be found: The El Greco fallacy in perception research. *Psychological Science,**25*(1), 38–46. 10.1177/0956797613485092 24297777 10.1177/0956797613485092

[CR99] Fischer, M. H., & Shaki, S. (2014). Spatial associations in numerical cognition—From single digits to arithmetic. *Quarterly Journal of Experimental Psychology,**67*(8), 1461–1483. 10.1080/17470218.2014.927515 10.1080/17470218.2014.927515 25051273

[CR100] Fiser, J., & Aslin, R. N. (2002). Statistical learning of higher-order temporal structure from visual shape sequences. *Journal of Experimental Psychology: Learning, Memory, and Cognition,**28*(3), 458–467. 10.1037/0278-7393.28.3.458 12018498 10.1037//0278-7393.28.3.458

[CR101] Fiser, J., & Aslin, R. N. (2002). Statistical learning of new visual feature combinations by infants. *Proceedings of the National Academy of Sciences of the United States of America,**99*(24), 15822–15826. 10.1073/pnas.232472899 12429858 10.1073/pnas.232472899 PMC137800

[CR102] Flusberg, S. J., Matlock, T., & Thibodeau, P. H. (2018). War metaphors in public discourse. *Metaphor and Symbol,**33*(1), 1–18. 10.1080/10926488.2018.1407992

[CR103] Fodor, J. A. (1983). *The modularity of mind*. MIT Press.

[CR104] Fotios, S., & Cheal, C. (2010). A comparison of simultaneous and sequential brightness judgements. *Lighting Research & Technology,**42*(2), 183–197. 10.1177/1477153509355506

[CR105] Frank, M. C., Everett, D. L., Fedorenko, E., & Gibson, E. (2008). Number as a cognitive technology: Evidence from Pirahã language and cognition. *Cognition,**108*(3), 819–824. 10.1016/j.cognition.2008.04.007 18547557 10.1016/j.cognition.2008.04.007

[CR106] Frank, M. C., Fedorenko, E., Lai, P., Saxe, R., & Gibson, E. (2012). Verbal interference suppresses exact numerical representation. *Cognitive Psychology,**64*(1), 74–92. 10.1016/j.cogpsych.2011.10.004 22112644 10.1016/j.cogpsych.2011.10.004

[CR107] Fuhrman, O., & Boroditsky, L. (2010). Cross-cultural differences in mental representations of time: Evidence from an implicit nonlinguistic task. *Cognitive Science,**34*(8), 1430–1451. 21564254 10.1111/j.1551-6709.2010.01105.x

[CR108] Fuhrman, O., McCormick, K., Chen, E., Jiang, H., Shu, D., Mao, S., & Boroditsky, L. (2011). How linguistic and cultural forces shape conceptions of time: English and Mandarin time in 3D. *Cognitive Science,**35*(7), 1305–1328. 21884222 10.1111/j.1551-6709.2011.01193.x

[CR109] Gallace, A., & Spence, C. (2006). Multisensory synesthetic interactions in the speeded classification of visual size. *Perception & Psychophysics,**68*(7), 1191–1203. 10.3758/BF03193720 17355042 10.3758/bf03193720

[CR110] Gao, C., & Shinkareva, S. V. (2021). Modality-general and modality-specific audiovisual valence processing. *Cortex,**138*, 127–137. 10.1016/j.cortex.2021.01.022 33684626 10.1016/j.cortex.2021.01.022

[CR111] Gendron, M., Lindquist, K. A., Barsalou, L., & Barrett, L. F. (2012). Emotion words shape emotion percepts. *Emotion,**12*(2), 314–325. 10.1037/a0026007 22309717 10.1037/a0026007 PMC4445832

[CR112] Gendron, M., Roberson, D., van der Vyver, J. M., & Barrett, L. F. (2014). Perceptions of emotion from facial expressions are not culturally universal: Evidence from a remote culture. *Emotion,**14*(2), 251–262. 10.1037/a0036052 24708506 10.1037/a0036052 PMC4752367

[CR113] Gentner, D. (1983). Structure-mapping: A theoretical framework for analogy. *Cognitive Science,**7*(2), 155–170.

[CR114] Gentner, D. (2010). Bootstrapping the mind: Analogical processes and symbol systems. *Cognitive Science,**34*(5), 752–775. 21564235 10.1111/j.1551-6709.2010.01114.x

[CR115] Gentner, D., & Christie, S. (2010). Mutual bootstrapping between language and analogical processing. *Language and Cognition,**2*(2), 261–283. 10.1515/langcog.2010.011

[CR116] Gentner, D., & Markman, A. B. (1997). Structure mapping in analogy and similarity. *American Psychologist,**52*(1), 45.

[CR117] Gentner, D., Özyürek, A., Gürcanli, Ö., & Goldin-Meadow, S. (2013). Spatial language facilitates spatial cognition: Evidence from children who lack language input. *Cognition,**127*(3), 318–330. 23542409 10.1016/j.cognition.2013.01.003 PMC3708650

[CR118] Ghazanfar, A. A., & Maier, J. X. (2009). Rhesus monkeys (*Macaca mulatta*) hear rising frequency sounds as looming. *Behavioral Neuroscience,**123*, 822–827. 10.1037/a0016391 19634941 10.1037/a0016391

[CR119] Ghazanfar, A. A., Turesson, H. K., Maier, J. X., Van Dinther, R., Patterson, R. D., & Logothetis, N. K. (2007). Vocal-tract resonances as indexical cues in rhesus monkeys. *Current Biology,**17*(5), 425–430. 10.1016/j.cub.2007.01.029 17320389 10.1016/j.cub.2007.01.029 PMC2361420

[CR120] Gibbs, R. (2006). Metaphor interpretation as embodied simulation. *Mind & Language,**21*(3), 434–458. 10.1111/j.1468-0017.2006.00285.x

[CR121] Gibbs, R. (2013). Walking the walk while thinking about the talk: Embodied interpretation of metaphorical narratives. *Journal of Psycholinguistic Research,**42*, 363–378. 22585389 10.1007/s10936-012-9222-6

[CR122] Gibbs, R., Gould, J. J., & Andric, M. (2006). Imagining metaphorical actions: Embodied simulations make the impossible plausible. *Imagination, Cognition and Personality,**25*(3), 221–238. 10.2190/97MK-44MV-1UUF-T5CR

[CR123] Giessner, S. R., Ryan, M. K., Schubert, T. W., & van Quaquebeke, N. (2011). The power of pictures: Vertical picture angles in power pictures. *Media Psychology,**14*(4), 442–464. 10.1080/15213269.2011.620541

[CR124] Giessner, S. R., & Schubert, T. W. (2007). High in the hierarchy: How vertical location and judgments of leaders’ power are interrelated. *Organizational Behavior and Human Decision Processes,**104*(1), 30–44. 10.1016/j.obhdp.2006.10.001

[CR125] Gijssels, T., Bottini, R., Rueschemeyer, S.-A., & Casasanto, D. (2013). Space and Time in the parietal cortex: fMRI evidence for a neural asymmetry. *Proceedings of the Annual Meeting of the Cognitive Science Society*, 7.

[CR126] Gilbert, A. N., Martin, R., & Kemp, S. E. (1996). Cross-modal correspondence between vision and olfaction: The color of smells. *The American Journal of Psychology*. 10.2307/1423010 8837406

[CR127] Glucksberg, S. (2008). How metaphors create categories—Quickly. In R. W. Gibbs Jr. (Ed.), *The Cambridge handbook of metaphor and thought* (pp. 67–83). Cambridge University Press. 10.1017/CBO9780511816802.006

[CR128] Goldstone, R. L., & Barsalou, L. W. (1998). Reuniting perception and conception. *Cognition,**65*(2), 231–262. 10.1016/S0010-0277(97)00047-4 9557384 10.1016/s0010-0277(97)00047-4

[CR129] Gottwald, J. M., Elsner, B., & Pollatos, O. (2015). Good is up—Spatial metaphors in action observation. *Frontiers in Psychology,**6*, Article 1605. 10.3389/fpsyg.2015.01605 26539147 10.3389/fpsyg.2015.01605 PMC4611084

[CR130] Goubet, N., Durand, K., Schaal, B., & McCall, D. D. (2018). Seeing odors in color: Cross-modal associations in children and adults from two cultural environments. *Journal of Experimental Child Psychology,**166*, 380–399. 10.1016/j.jecp.2017.09.007 29028585 10.1016/j.jecp.2017.09.007

[CR131] Grossmann, T. (2010). The development of emotion perception in face and voice during infancy. *Restorative Neurology and Neuroscience,**28*(2), 219–236. 10.3233/RNN-2010-0499 20404410 10.3233/RNN-2010-0499

[CR132] Guedes, D., Vaz Garrido, M., Lamy, E., Pereira Cavalheiro, B., & Prada, M. (2023). Crossmodal interactions between audition and taste: A systematic review and narrative synthesis. *Food Quality and Preference,**107*, Article 104856. 10.1016/j.foodqual.2023.104856

[CR133] Hafri, A., & Firestone, C. (2021). The perception of relations. *Trends in Cognitive Sciences,**25*(6), 475–492. 10.1016/j.tics.2021.01.006 33812770 10.1016/j.tics.2021.01.006

[CR134] Hanson-Vaux, G., Crisinel, A.-S., & Spence, C. (2013). Smelling shapes: Crossmodal correspondences between odors and shapes. *Chemical Senses,**38*(2), 161–166. 10.1093/chemse/bjs087 23118203 10.1093/chemse/bjs087

[CR135] Harlow, H. F. (1958). The nature of love. *American Psychologist,**13*(12), 673–685. 10.1037/h0047884

[CR136] Harnad, S. (2005). Cognition is categorization. In H. Cohen & C. Lefebvre (Eds.), *Handbook of categorization in cognitive science* (pp. 20–45). Elsevier Science.

[CR137] Hartmann, M., & Mast, F. W. (2017). Loudness counts: Interactions between loudness, number magnitude, and space. *Quarterly Journal of Experimental Psychology,**70*(7), 1305–1322. 10.1080/17470218.2016.1182194 10.1080/17470218.2016.1182194 27109592

[CR138] Hebb, D. O. (1949). *The organization of behavior: A neuropsychological theory*. Wiley.

[CR139] Hendricks, R. K., Demjén, Z., Semino, E., & Boroditsky, L. (2018). Emotional implications of metaphor: Consequences of metaphor framing for mindset about cancer. *Metaphor and Symbol,**33*(4), 267–279.

[CR140] Holler, J., Drijvers, L., Rafiee, A., & Majid, A. (2022). Embodied space–pitch associations are shaped by language. *Cognitive Science,**46*(2), Article e13083. 10.1111/cogs.13083 35188682 10.1111/cogs.13083

[CR141] Holmes, K. J., Flusberg, S. J., & Thibodeau, P. H. (2018). Compound words reflect cross-culturally shared bodily metaphors. *Cognitive Science,**42*(8), 3071–3082. 10.1111/cogs.12671 30109729 10.1111/cogs.12671

[CR142] Holmes, K. J., & Lourenco, S. F. (2011). Common spatial organization of number and emotional expression: A mental magnitude line. *Brain and Cognition,**77*(2), 315–323. 10.1016/j.bandc.2011.07.002 21839568 10.1016/j.bandc.2011.07.002

[CR143] Hommel, B., Müsseler, J., Aschersleben, G., & Prinz, W. (2001). The theory of event coding (TEC): A framework for perception and action planning. *Behavioral and Brain Sciences,**24*(5), 849–878. 10.1017/S0140525X01000103 12239891 10.1017/s0140525x01000103

[CR144] Horchak, O., Giger, J.-C., & Pochwatko, G. (2014). Simulation of metaphorical actions and discourse comprehension. *Metaphor and Symbol*. 10.1080/10926488.2014.859045

[CR145] Huang, Y., & Tse, C.-S. (2015). Re-examining the automaticity and directionality of the activation of the spatial-valence “good is up” metaphoric association. *PLoS One,**10*(4), Article e0123371. 10.1371/journal.pone.0123371 25867748 10.1371/journal.pone.0123371 PMC4395106

[CR146] Hubbard, E. M., Arman, A. C., Ramachandran, V. S., & Boynton, G. M. (2005). Individual differences among grapheme-color synesthetes: Brain-behavior correlations. *Neuron,**45*(6), 975–985. 10.1016/j.neuron.2005.02.008 15797557 10.1016/j.neuron.2005.02.008

[CR147] IJzerman, H., & Semin, G. R. (2009). The thermometer of social relations: Mapping social proximity on temperature. *Psychological Science,**20*(10), 1214–1220. 10.1111/j.1467-9280.2009.02434.x 19732385 10.1111/j.1467-9280.2009.02434.x

[CR148] Inagaki, T. K., & Eisenberger, N. I. (2013). Shared neural mechanisms underlying social warmth and physical warmth. *Psychological Science,**24*(11), 2272–2280. 10.1177/0956797613492773 24048423 10.1177/0956797613492773

[CR149] Ishihara, M., Keller, P. E., Rossetti, Y., & Prinz, W. (2008). Horizontal spatial representations of time: Evidence for the STEARC effect. *Cortex,**44*(4), 454–461. 10.1016/j.cortex.2007.08.010 18387578 10.1016/j.cortex.2007.08.010

[CR150] Ito, Y., & Hatta, T. (2004). Spatial structure of quantitative representation of numbers: Evidence from the SNARC effect. *Memory & Cognition,**32*(4), 662–673. 10.3758/BF03195857 15478760 10.3758/bf03195857

[CR151] Jackson, J. C., Watts, J., Henry, T. R., List, J.-M., Forkel, R., Mucha, P. J., Greenhill, Simon J.., Gray, Russell D.., & Lindquist, K. A. (2019). Emotion semantics show both cultural variation and universal structure. *Science,**366*(6472), 1517–1522. 10.1126/science.aaw8160 31857485 10.1126/science.aaw8160

[CR152] James, W. (1890). *The principles of psychology.* Henry Holt.

[CR153] Johansson, R. C. G., Kelber, P., & Ulrich, R. (2024). Speeded classification of visual events is sensitive to crossmodal intensity correspondence. *Journal of Experimental Psychology. Human Perception and Performance*. 10.1037/xhp0001183 38546625 10.1037/xhp0001183

[CR154] Jonauskaite, D., Abu-Akel, A., Dael, N., Oberfeld, D., Abdel-Khalek, A. M., Al-Rasheed, A. S., Antonietti, Jean-Philippe., Bogushevskaya, Victoria, Chamseddine, Amer, Chkonia, Eka, Corona, Violeta, Fonseca-Pedrero, Eduardo, Griber, Yulia A.., Grimshaw, Gina, Hasan, Aya Ahmed, Havelka, Jelena, Hirnstein, Marco, Karlsson, Bodil S. A.., Laurent, Eric, … Mohr, C. (2020). Universal patterns in color-emotion associations are further shaped by linguistic and geographic proximity. *Psychological Science,**31*(10), 1245–1260. 10.1177/0956797620948810 32900287 10.1177/0956797620948810

[CR155] Jostmann, N. B., Lakens, D., & Schubert, T. W. (2009). Weight as an embodiment of importance. *Psychological Science,**20*(9), 1169–1174. 10.1111/j.1467-9280.2009.02426.x 19686292 10.1111/j.1467-9280.2009.02426.x

[CR156] Karwoski, T. F., Odbert, H. S., & Osgood, C. E. (1942). Studies in synesthetic thinking: II. The role of form in visual responses to music. *The Journal of General Psychology,**26*(2), 199–222.

[CR157] Keysar, B. (1989). On the functional equivalence of literal and metaphorical interpretations in discourse. *Journal of Memory and Language,**28*(4), 375–385. 10.1016/0749-596X(89)90017-X

[CR158] Khatin-Zadeh, O., Farsani, D., Hu, J., Eskandari, Z., Zhu, Y., & Banaruee, H. (2023). A review of studies supporting metaphorical embodiment. *Behavioral Sciences,**13*(7), 585. 37504032 10.3390/bs13070585 PMC10376178

[CR159] Kim, J. S., Aheimer, B., Montané Manrara, V., & Bedny, M. (2021). Shared understanding of color among sighted and blind adults. *Proceedings of the National Academy of Sciences of the United States of America,**118*(33), Article e2020192118. 10.1073/pnas.2020192118 34385310 10.1073/pnas.2020192118 PMC8379969

[CR160] Kim, Y.-J. (2008). Cross-modal associations between colors and fragrances for commercial perfume design. *Science of Emotion and Sensibility,**11*(3), 427–439.

[CR161] Kim, Y.-J. (2013). Can eyes smell? Cross-modal correspondences between color hue-tone and fragrance family. *Color Research and Application,**38*(2), 139–156. 10.1002/col.20717

[CR162] Klooster, N., McQuire, M., Grossman, M., McMillan, C., Chatterjee, A., & Cardillo, E. (2020). The neural basis of metaphor comprehension: Evidence from left hemisphere degeneration. *Neurobiology of Language,**1*(4), 474–491. 10.1162/nol_a_00022 37215584 10.1162/nol_a_00022 PMC10158586

[CR163] Knöferle, K., & Spence, C. (2012). Crossmodal correspondences between sounds and tastes. *Psychonomic Bulletin & Review*. 10.3758/s13423-012-0321-z 10.3758/s13423-012-0321-z 23055144

[CR164] Kominsky, J. F., & Scholl, B. J. (2020). Retinotopic adaptation reveals distinct categories of causal perception. *Cognition,**203*, Article 104339. 10.1016/j.cognition.2020.104339 32711120 10.1016/j.cognition.2020.104339 PMC7484022

[CR165] Korzeniowska, A. T., Root-Gutteridge, H., Simner, J., & Reby, D. (2019). Audio–visual crossmodal correspondences in domestic dogs (*Canis familiaris*). *Biology Letters,**15*(11), Article 20190564. 10.1098/rsbl.2019.0564 31718513 10.1098/rsbl.2019.0564 PMC6892510

[CR166] Lacey, S., Stilla, R., & Sathian, K. (2012). Metaphorically feeling: Comprehending textural metaphors activates somatosensory cortex. *Brain and Language,**120*(3), 416–421. 10.1016/j.bandl.2011.12.016 22305051 10.1016/j.bandl.2011.12.016 PMC3318916

[CR167] Lakens, D. (2012). Polarity correspondence in metaphor congruency effects: Structural overlap predicts categorization times for bipolar concepts presented in vertical space. *Journal of Experimental Psychology: Learning, Memory, and Cognition,**38*(3), 726–736. 10.1037/a0024955 21843022 10.1037/a0024955

[CR168] Lakens, D., Semin, G. R., & Foroni, F. (2012). But for the bad, there would not be good: Grounding valence in brightness through shared relational structures. *Journal of Experimental Psychology: General*. 10.1037/a0026468 22201411 10.1037/a0026468

[CR169] Lakoff, G. (2009). *The neural theory of metaphor*. SSRN 1437794.

[CR170] Lakoff, G., & Johnson, M. (1980). Conceptual metaphor in everyday language. *Journal of Philosophy,**77*(8), 453–486. 10.2307/2025464

[CR171] Lakoff, G., & Johnson, M. (1980). The metaphorical structure of the human conceptual system. *Cognitive Science,**4*(2), 195–208.

[CR172] Lakoff, G., & Johnson, M. (1999). *Philosophy in the flesh: The embodied mind and its challenge to western thought* (Vol. 640). Basic Books.

[CR173] Lebrecht, S., Bar, M., Barrett, L., & Tarr, M. (2012). Micro-valences: Perceiving affective valence in everyday objects. *Frontiers in Psychology*, *3*. https://www.frontiersin.org/article/10.3389/fpsyg.2012.00107 10.3389/fpsyg.2012.00107 PMC3328080 22529828

[CR174] LeDoux, J. (2003). The emotional brain, fear, and the amygdala. *Cellular and Molecular Neurobiology,**23*(4), 727–738. 10.1023/A:1025048802629 14514027 10.1023/A:1025048802629 PMC11530156

[CR175] Lee, S. W. S., & Schwarz, N. (2010). Dirty hands and dirty mouths: Embodiment of the moral-purity metaphor is specific to the motor modality involved in moral transgression. *Psychological Science,**21*(10), 1423–1425. 10.1177/0956797610382788 20817782 10.1177/0956797610382788

[CR176] Lenci, A., Baroni, M., Cazzolli, G., & Marotta, G. (2013). Blind: A set of semantic feature norms from the congenitally blind. *Behavior Research Methods,**45*(4), 1218–1233. 10.3758/s13428-013-0323-4 23435658 10.3758/s13428-013-0323-4

[CR177] Levitan, C. A., Ren, J., Woods, A. T., Boesveldt, S., Chan, J. S., McKenzie, K. J., Dodson, Michael, Levin, Jai A.., Leong, Christine X. R.., & van den Bosch, J. J. F. (2014). Cross-cultural color-odor associations. *PLoS One,**9*(7), Article e101651. 10.1371/journal.pone.0101651 25007343 10.1371/journal.pone.0101651 PMC4089998

[CR178] Lewkowicz, D. J., & Turkewitz, G. (1980). Cross-modal equivalence in early infancy: Auditory–visual intensity matching. *Developmental Psychology,**16*(6), 597.

[CR179] Lievers, F. S. (2017). Figures and the senses: Towards a definition of synaesthesia. *Review of Cognitive Linguistics,**15*(1), 83–101. 10.1075/rcl.15.1.04str

[CR180] Lindemann, O., Abolafia, J., Girardi, G., & Bekkering, H. (2008). Getting a grip on numbers: Numerical magnitude priming in object grasping. *Journal of Experimental Psychology. Human Perception and Performance,**33*, 1400–1409. 10.1037/0096-1523.33.6.1400 10.1037/0096-1523.33.6.1400 18085952

[CR181] Lindquist, K. A., Barrett, L. F., Bliss-Moreau, E., & Russell, J. A. (2006). Language and the perception of emotion. *Emotion,**6*(1), 125–138. 10.1037/1528-3542.6.1.125 16637756 10.1037/1528-3542.6.1.125

[CR182] Lindquist, K. A., & Gendron, M. (2013). What’s in a word? Language constructs emotion perception. *Emotion Review,**5*(1), 66–71. 10.1177/1754073912451351

[CR183] Lindquist, K. A., Gendron, M., Barrett, L. F., & Dickerson, B. C. (2014). Emotion perception, but not affect perception, is impaired with semantic memory loss. *Emotion,**14*(2), 375–387. 10.1037/a0035293 24512242 10.1037/a0035293 PMC4119962

[CR184] Lindquist, K. A., MacCormack, J. K., & Shablack, H. (2015). The role of language in emotion: Predictions from psychological constructionism. *Frontiers in Psychology,**6*, Article 444. 10.3389/fpsyg.2015.00444 25926809 10.3389/fpsyg.2015.00444 PMC4396134

[CR185] Lindquist, K. A., Siegel, E. H., Quigley, K. S., & Barrett, L. F. (2013). The hundred-year emotion war: Are emotions natural kinds or psychological constructions? Comment on Lench, Flores, and Bench (2011). *Psychological Bulletin,**139*(1), 255–263. 10.1037/a0029038 23294094 10.1037/a0029038 PMC3556454

[CR186] Liu, Q., Ham, H., Cao, K., & Lupyan, G. (2024). Why is Bach blue instead of red? Different strategies moderate people’s color-music associations. *Proceedings of the Annual Meeting of the Cognitive Science Society*, *46*(0). https://escholarship.org/uc/item/13n3w8vr

[CR187] Liu, Q., & Lupyan, G. (2023). Cross-domain semantic alignment: Concrete concepts are more abstract than you think. *Philosophical Transactions of the Royal Society B: Biological Sciences,**378*(1870), 20210372. 10.1098/rstb.2021.0372 10.1098/rstb.2021.0372 PMC9791493 36571138

[CR188] Loewenstein, J., & Gentner, D. (2005). Relational language and the development of relational mapping. *Cognitive Psychology,**50*(4), 315–353. 15893523 10.1016/j.cogpsych.2004.09.004

[CR189] Lourenco, S. F., & Longo, M. R. (2010). General magnitude representation in human infants. *Psychological Science,**21*(6), 873–881. 10.1177/0956797610370158 20431048 10.1177/0956797610370158 PMC2930776

[CR190] Lourenco, S. F., & Longo, M. R. (2011). Origins and development of generalized magnitude representation. In S. Dehaene & E. M. Brannon (Eds.), *Space, time and number in the brain* (pp. 225–244). Elsevier. 10.1016/B978-0-12-385948-8.00015-3

[CR191] Ludwig, V. U., Adachi, I., & Matsuzawa, T. (2011). Visuoauditory mappings between high luminance and high pitch are shared by chimpanzees (*Pan troglodytes*) and humans. *Proceedings of the National Academy of Sciences of the United States of America,**108*(51), 20661–20665. 22143791 10.1073/pnas.1112605108 PMC3251154

[CR192] Lupyan, G. (2015). Cognitive penetrability of perception in the age of prediction: Predictive systems are penetrable systems. *Review of Philosophy and Psychology,**6*(4), 547–569.

[CR193] Lupyan, G. (2017). Objective effects of knowledge on visual perception. *Journal of Experimental Psychology: Human Perception and Performance,**43*(4), 794. 28345946 10.1037/xhp0000343

[CR194] Lupyan, G., Abdel Rahman, R., Boroditsky, L., & Clark, A. (2020). Effects of language on visual perception. *Trends in Cognitive Sciences,**24*(11), 930–944. 10.1016/j.tics.2020.08.005 33012687 10.1016/j.tics.2020.08.005

[CR195] Lupyan, G., & Winter, B. (2018). Language is more abstract than you think, or, Why aren’t languages more iconic? *Philosophical Transactions of the Royal Society B: Biological Sciences,**373*(1752), Article 20170137. 10.1098/rstb.2017.0137 PMC6015821 29915005

[CR196] Lynott, D., Corker, K., Connell, L., & O’Brien, K. (2023). The effects of temperature on prosocial and antisocial behaviour: A review and meta-analysis. *British Journal of Social Psychology,**62*(3), 1177–1214. 10.1111/bjso.12626 36794795 10.1111/bjso.12626

[CR197] Lynott, D., & Coventry, K. (2014). On the ups and downs of emotion: Testing between conceptual-metaphor and polarity accounts of emotional valence–spatial location interactions. *Psychonomic Bulletin & Review,**21*(1), 218–226. 10.3758/s13423-013-0481-5 23904350 10.3758/s13423-013-0481-5

[CR198] Mandler, J. M. (1992). How to build a baby: II. Conceptual primitives. *Psychological Review,**99*(4), 587–604. 10.1037/0033-295X.99.4.587 1454900 10.1037/0033-295x.99.4.587

[CR199] Marks, L. E. (1974). On associations of light and sound: The mediation of brightness, pitch, and loudness. *The American Journal of Psychology*. 10.2307/1422011 4451203

[CR200] Marks, L. E. (1975). On colored-hearing synesthesia: Cross-modal translations of sensory dimensions. *Psychological Bulletin,**82*(3), 303–331. 10.1037/0033-2909.82.3.303 1096209

[CR201] Marks, L. E. (1987). On cross-modal similarity: Auditory–visual interactions in speeded discrimination. *Journal of Experimental Psychology. Human Perception and Performance,**13*(3), 384. 2958587 10.1037//0096-1523.13.3.384

[CR202] Marks, L. E. (1988). Magnitude estimation and sensory matching. *Perception & Psychophysics,**43*(6), 511–525. 10.3758/BF03207739 3399349 10.3758/bf03207739

[CR203] Martino, G., & Marks, L. E. (1999). Perceptual and linguistic interactions in speeded classification: Tests of the semantic coding hypothesis. *Perception,**28*(7), 903–923. 10664781 10.1068/p2866

[CR204] Martino, G., & Marks, L. E. (2000). Cross-modal interaction between vision and touch: The role of synesthetic correspondence. *Perception,**29*(6), 745–754. 10.1068/p2984 11040956 10.1068/p2984

[CR205] Matlock, T., Holmes, K. J., Srinivasan, M., & Ramscar, M. (2011). Even abstract motion influences the understanding of time. *Metaphor and Symbol,**26*(4), 260–271. 10.1080/10926488.2011.609065

[CR206] McCormick, K., Lacey, S., Stilla, R., Nygaard, L. C., & Sathian, K. (2021). Neural basis of the sound-symbolic crossmodal correspondence between auditory pseudowords and visual shapes. *Multisensory Research,**35*(1), 29–78. 10.1163/22134808-bja10060 34384048 10.1163/22134808-bja10060 PMC9196751

[CR207] McGlone, M. S., & Manfredi, D. A. (2001). Topic—Vehicle interaction in metaphor comprehension. *Memory & Cognition,**29*(8), 1209–1219. 10.3758/BF03206390 11913757 10.3758/bf03206390

[CR208] McGurk, H., & Macdonald, J. (1976). Hearing lips and seeing voices. *Nature*. 10.1038/264746a0 1012311 10.1038/264746a0

[CR209] Meier, B., & Dionne, S. (2009). Downright sexy: Verticality, implicit power, and perceived physical attractiveness. *Social Cognition,**27*(6), 883–892. 10.1521/soco.2009.27.6.883

[CR210] Meier, B., Robinson, M., Crawford, L. E., & Ahlvers, W. (2007). When “light” and “dark” thoughts become light and dark responses: Affect biases brightness judgments. *Emotion,**7*, 366–376. 10.1037/1528-3542.7.2.366 17516814 10.1037/1528-3542.7.2.366

[CR211] Meier, B., Robinson, M. D., & Clore, G. L. (2004). Why good guys wear white: Automatic inferences about stimulus valence based on brightness. *Psychological Science,**15*(2), 82–87. 10.1111/j.0963-7214.2004.01502002.x 14738513 10.1111/j.0963-7214.2004.01502002.x

[CR212] Meier, B. P., & Fetterman, A. K. (2022). Metaphors for god: God is high, bright, and human in implicit tasks. *Psychology of Religion and Spirituality,**14*(1), 43–50. 10.1037/rel0000324

[CR213] Meier, B. P., Hauser, D. J., Robinson, M. D., Friesen, C. K., & Schjeldahl, K. (2007). What’s “up” with God? Vertical space as a representation of the divine. *Journal of Personality and Social Psychology,**93*(5), 699–710. 10.1037/0022-3514.93.5.699 17983295 10.1037/0022-3514.93.5.699

[CR214] Meier, B. P., Sellbom, M., & Wygant, D. B. (2007). Failing to take the moral high ground: Psychopathy and the vertical representation of morality. *Personality and Individual Differences,**43*(4), 757–767. 10.1016/j.paid.2007.02.001

[CR215] Melara, R. D. (1989). Dimensional interaction between color and pitch. *Journal of Experimental Psychology. Human Perception and Performance,**15*(1), Article 69. 2522534 10.1037//0096-1523.15.1.69

[CR216] Melara, R. D., & Marks, L. E. (1990). Interaction among auditory dimensions: Timbre, pitch, and loudness. *Perception & Psychophysics,**48*(2), 169–178. 10.3758/BF03207084 2385491 10.3758/bf03207084

[CR217] Melara, R. D., & O’Brien, T. P. (1987). Interaction between synesthetically corresponding dimensions. *Journal of Experimental Psychology: General,**116*(4), 323.

[CR218] Merritt, D. J., Casasanto, D., & Brannon, E. M. (2010). Do monkeys think in metaphors? Representations of space and time in monkeys and humans. *Cognition,**117*(2), 191–202. 10.1016/j.cognition.2010.08.011 20846645 10.1016/j.cognition.2010.08.011 PMC2952654

[CR219] Milhau, A., Brouillet, T., Dru, V., Coello, Y., & Brouillet, D. (2017). Valence activates motor fluency simulation and biases perceptual judgment. *Psychological Research,**81*(4), 795–805. 10.1007/s00426-016-0788-8 27417215 10.1007/s00426-016-0788-8

[CR220] Miskovic, V., & Anderson, A. (2018). Modality general and modality specific coding of hedonic valence. *Current Opinion in Behavioral Sciences,**19*, 91–97. 10.1016/j.cobeha.2017.12.012 29967806 10.1016/j.cobeha.2017.12.012 PMC6024250

[CR221] Mondloch, C. J., & Maurer, D. (2004). Do small white balls squeak? Pitch-object correspondences in young children. *Cognitive, Affective, & Behavioral Neuroscience,**4*(2), 133–136. 10.3758/CABN.4.2.133 10.3758/cabn.4.2.133 15460920

[CR222] Monetta, L., & Pell, M. D. (2007). Effects of verbal working memory deficits on metaphor comprehension in patients with Parkinson’s disease. *Brain and Language,**101*(1), 80–89. 10.1016/j.bandl.2006.06.007 16875726 10.1016/j.bandl.2006.06.007

[CR223] Motoki, K., Spence, C., & Velasco, C. (2024). Colour/shape-taste correspondences across three languages in chatGPT. *Cognition,**253*, Article 105936. 10.1016/j.cognition.2024.105936 39217782 10.1016/j.cognition.2024.105936

[CR224] Motoki, K., Takahashi, N., Velasco, C., & Spence, C. (2022). Is classical music sweeter than jazz? Crossmodal influences of background music and taste/flavour on healthy and indulgent food preferences. *Food Quality and Preference,**96*, Article 104380. 10.1016/j.foodqual.2021.104380

[CR225] Motoki, K., & Velasco, C. (2021). Taste-shape correspondences in context. *Food Quality and Preference,**88*, Article 104082. 10.1016/j.foodqual.2020.104082

[CR226] Niedeggen, M., Kerschreiter, R., Hirte, D., & Weschke, S. (2017). Being low prepares for being neglected: Verticality affects expectancy of social participation. *Psychonomic Bulletin & Review,**24*(2), 574–581. 10.3758/s13423-016-1115-5 27368640 10.3758/s13423-016-1115-5

[CR227] Nook, E. C., Sasse, S. F., Lambert, H. K., McLaughlin, K. A., & Somerville, L. H. (2017). Increasing verbal knowledge mediates development of multidimensional emotion representations. *Nature Human Behaviour,**1*(12), 881–889. 10.1038/s41562-017-0238-7 29399639 10.1038/s41562-017-0238-7 PMC5790154

[CR228] Nosofsky, R. M. (2011). The generalized context model: An exemplar model of classification. In E. M. Pothos & A. J. Wills (Eds.), *Formal approaches in categorization* (pp. 18–39). Cambridge University Press. 10.1017/CBO9780511921322.002

[CR229] Núñez, R., & Cooperrider, K. (2013). The tangle of space and time in human cognition. *Trends in Cognitive Sciences,**17*(5), 220–229. 10.1016/j.tics.2013.03.008 23608363 10.1016/j.tics.2013.03.008

[CR230] Núñez, R. E., & Sweetser, E. (2006). With the future behind them: Convergent evidence from Aymara language and gesture in the crosslinguistic comparison of spatial construals of time. *Cognitive Science,**30*(3), 401–450. 10.1207/s15516709cog0000_62 21702821 10.1207/s15516709cog0000_62

[CR231] Occelli, V., Spence, C., & Zampini, M. (2009). Compatibility effects between sound frequency and tactile elevation. *Neuroreport,**20*(8), 793–797. 10.1097/WNR.0b013e32832b8069 19369906 10.1097/WNR.0b013e32832b8069

[CR232] Ohla, K., Toepel, U., le Coutre, J., & Hudry, J. (2012). Visual-gustatory interaction: Orbitofrontal and insular cortices mediate the effect of high-calorie visual food cues on taste pleasantness. *PLoS One,**7*(3), Article e32434. 10.1371/journal.pone.0032434 22431974 10.1371/journal.pone.0032434 PMC3303800

[CR233] Okubo, M., & Ishikawa, K. (2011). Automatic semantic association between emotional valence and brightness in the right hemisphere. *Cognition and Emotion,**25*(7), 1273–1280. 10.1080/02699931.2010.541658 21432631 10.1080/02699931.2010.541658

[CR234] OpenAI. (2023). *ChatGPT* (Mar 14 version) [Large language model; Computer software]. https://chat.openai.com/chat

[CR235] Osgood, C. E., Suci, G. J., & Tannenbaum, P. H. (1957). *The measurement of meaning*. University of Illinois Press.

[CR236] Ozturk, O., Krehm, M., & Vouloumanos, A. (2013). Sound symbolism in infancy: Evidence for sound–shape cross-modal correspondences in 4-month-olds. *Journal of Experimental Child Psychology,**114*(2), 173–186. 10.1016/j.jecp.2012.05.004 22960203 10.1016/j.jecp.2012.05.004

[CR237] Palmer, S. E., Langlois, T. A., & Schloss, K. B. (2016). Music-to-color associations of single-line piano melodies in non-synesthetes. *Multisensory Research,**29*(1/3), 157–193. 27311295 10.1163/22134808-00002486

[CR238] Palmer, S. E., & Schloss, K. B. (2010). An ecological valence theory of human color preference. *Proceedings of the National Academy of Sciences of the United States of America,**107*(19), 8877–8882. 10.1073/pnas.0906172107 20421475 10.1073/pnas.0906172107 PMC2889342

[CR239] Palmer, S. E., Schloss, K. B., Xu, Z., & Prado-León, L. R. (2013). Music–color associations are mediated by emotion. *Proceedings of the National Academy of Sciences of the United States of America,**110*(22), 8836–8841. 10.1073/pnas.1212562110 23671106 10.1073/pnas.1212562110 PMC3670360

[CR240] Papagno, C. (2001). Comprehension of metaphors and idioms in patients with Alzheimer’s disease: A longitudinal study. *Brain,**124*(7), 1450–1460. 10.1093/brain/124.7.1450 11408339 10.1093/brain/124.7.1450

[CR241] Parise, C. V., Knorre, K., & Ernst, M. O. (2014). Natural auditory scene statistics shapes human spatial hearing. *Proceedings of the National Academy of Sciences of the United States of America,**111*(16), 6104–6108. 10.1073/pnas.1322705111 24711409 10.1073/pnas.1322705111 PMC4000839

[CR242] Parise, C. V., & Spence, C. (2008). Synesthetic congruency modulates the temporal ventriloquism effect. *Neuroscience Letters,**442*(3), 257–261. 18638522 10.1016/j.neulet.2008.07.010

[CR243] Parise, C. V., & Spence, C. (2009). ‘When birds of a feather flock together’: Synesthetic correspondences modulate audiovisual integration in non-synesthetes. *PLoS One,**4*(5), Article e5664. 10.1371/journal.pone.0005664 19471644 10.1371/journal.pone.0005664 PMC2680950

[CR244] Parkinson, C., Kohler, P. J., Sievers, B., & Wheatley, T. (2012). Associations between auditory pitch and visual elevation do not depend on language: Evidence from a remote population. *Perception,**41*(7), 854–861. 10.1068/p7225 23155736 10.1068/p7225

[CR245] Perlman, M., Dale, R., & Lupyan, G. (2014). Iterative vocal charades: The emergence of conventions in vocal communication. *Evolution of Language: Proceedings of the 10th International Conference (EVOLANG10)*, 236–243.

[CR246] Perniss, P., Thompson, R., & Vigliocco, G. (2010). Iconicity as a general property of language: Evidence from spoken and signed languages. *Frontiers in Psychology*, *0*. 10.3389/fpsyg.2010.00227 10.3389/fpsyg.2010.00227 PMC3153832 21833282

[CR247] Pinel, P., Piazza, M., Le Bihan, D., & Dehaene, S. (2004). Distributed and overlapping cerebral representations of number, size, and luminance during comparative judgments. *Neuron,**41*(6), 983–993. 10.1016/s0896-6273(04)00107-2 15046729 10.1016/s0896-6273(04)00107-2

[CR248] Pitt, B., & Casasanto, D. (2018). Spatializing emotion: No evidence for a domain-general magnitude system. *Cognitive Science,**42*(7), 2150–2180. 10.1111/cogs.12568 29164659 10.1111/cogs.12568

[CR249] Pitt, B., Gibson, E., & Piantadosi, S. T. (2022). Exact number concepts are limited to the verbal count range. *Psychological Science,**33*(3), 371–381. 10.1177/09567976211034502 35132893 10.1177/09567976211034502 PMC9096449

[CR250] Pramudya, R. C., Choudhury, D., Zou, M., & Seo, H.-S. (2020). “Bitter touch”: Cross-modal associations between hand-feel touch and gustatory cues in the context of coffee consumption experience. *Food Quality and Preference,**83*, Article 103914. 10.1016/j.foodqual.2020.103914

[CR251] Puigcerver, L., Rodríguez-Cuadrado, S., Gómez-Tapia, V., & Navarra, J. (2019). Vertical mapping of auditory loudness: Loud is high, but quiet is not always low. *Psicológica Journal,**40*(2), 85–104. 10.2478/psicolj-2019-0006

[CR252] Pylyshyn, Z. (1999). Is vision continuous with cognition?: The case for cognitive impenetrability of visual perception. *Behavioral and Brain Sciences,**22*(3), 341–365. 10.1017/S0140525X99002022 11301517 10.1017/s0140525x99002022

[CR253] Quinn, P. C., Anzures, G., Izard, C. E., Lee, K., Pascalis, O., Slater, A. M., & Tanaka, J. W. (2011). Looking across domains to understand infant representation of emotion. *Emotion Review,**3*(2), 197–206. 10.1177/1754073910387941 21572929 10.1177/1754073910387941 PMC3092399

[CR254] Quinn, P. C., & Eimas, P. D. (1997). A reexamination of the perceptual-to-conceptual shift in mental representations. *Review of General Psychology,**1*(3), 271–287. 10.1037/1089-2680.1.3.271

[CR255] Radford, A., Wu, J., Child, R., Luan, D., Amodei, D., & Sutskever, I. (2019). Language models are unsupervised multitask learners. *OpenAI Blog,**1*(8), 9.

[CR256] Ramachandran, V. S., & Brang, D. (2008). Tactile-emotion synesthesia. *Neurocase,**14*(5), 390–399. 18821168 10.1080/13554790802363746

[CR257] Ramachandran, V. S., & Hubbard, E. M. (2001). Synaesthesia—a window into perception, thought and language. *Journal of Consciousness Studies,**8*(12), 3–34.

[CR258] Reber, R., Winkielman, P., & Schwarz, N. (1998). Effects of perceptual fluency on affective judgments. *Psychological Science,**9*(1), 45–48. 10.1111/1467-9280.00008

[CR259] Reilly, M., Howerton, O., & Desai, R. H. (2019). Time-course of motor involvement in literal and metaphoric action sentence processing: A TMS study. *Frontiers in Psychology*. 10.3389/fpsyg.2019.00371 30863346 10.3389/fpsyg.2019.00371 PMC6399124

[CR260] Rissman, L., Liu, Q., & Lupyan, G. (2023). Gaps in the lexicon restrict communication. *Open Mind,**7*, 412–434. 10.1162/opmi_a_00089 37637298 10.1162/opmi_a_00089 PMC10449401

[CR261] Ro, T., Hsu, J., Yasar, N. E., Caitlin Elmore, L., & Beauchamp, M. S. (2009). Sound enhances touch perception. *Experimental Brain Research,**195*(1), 135–143. 10.1007/s00221-009-1759-8 19305983 10.1007/s00221-009-1759-8

[CR262] Rolfs, M., Dambacher, M., & Cavanagh, P. (2013). Visual adaptation of the perception of causality. *Current Biology,**23*(3), 250–254. 10.1016/j.cub.2012.12.017 23313360 10.1016/j.cub.2012.12.017

[CR263] Root-Bernstein, R., & Root-Bernstein, M. (1999). *Sparks of genius: The thirteen thinking tools of creative people* (pp. x–377). Houghton Mifflin.

[CR264] Ruba, A. L., Meltzoff, A. N., & Repacholi, B. M. (2019). How do you feel? Preverbal infants match negative emotions to events. *Developmental Psychology,**55*(6), 1138–1149. 10.1037/dev0000711 30829508 10.1037/dev0000711

[CR265] Rugani, R., Kelly, D. M., Szelest, I., Regolin, L., & Vallortigara, G. (2010). Is it only humans that count from left to right? *Biology Letters,**6*(3), 290–292. 10.1098/rsbl.2009.0960 20071393 10.1098/rsbl.2009.0960 PMC2880063

[CR266] Rugani, R., Vallortigara, G., Priftis, K., & Regolin, L. (2015). Animal cognition. Number-space mapping in the newborn chick resembles humans’ mental number line. *Science (New York, N.Y.),**347*(6221), 534–536. 10.1126/science.aaa1379 25635096 10.1126/science.aaa1379

[CR267] Rusconi, E., Kwan, B., Giordano, B. L., Umiltà, C., & Butterworth, B. (2006). Spatial representation of pitch height: The SMARC effect. *Cognition,**99*(2), 113–129. 10.1016/j.cognition.2005.01.004 15925355 10.1016/j.cognition.2005.01.004

[CR268] Salgado Montejo, A., Alvarado, J., Velasco, C., Salgado, C., Hasse, K., & Spence, C. (2015). The sweetest thing: The influence of angularity, symmetry, and the number of elements on shape-valence and shape-taste matches. *Frontiers in Psychology,**6*, Article 1382. 10.3389/fpsyg.2015.01382 26441757 10.3389/fpsyg.2015.01382 PMC4569812

[CR269] Saluja, S., & Stevenson, R. J. (2018). Cross-modal associations between real tastes and colors. *Chemical Senses,**43*(7), 475–480. 10.1093/chemse/bjy033 29868904 10.1093/chemse/bjy033

[CR270] Santiago, J., & Lakens, D. (2015). Can conceptual congruency effects between number, time, and space be accounted for by polarity correspondence? *Acta Psychologica,**156*, 179–191. 10.1016/j.actpsy.2014.09.016 25542784 10.1016/j.actpsy.2014.09.016

[CR271] Santiago, J., Lupáñez, J., Pérez, E., & Funes, M. J. (2007). Time (also) flies from left to right. *Psychonomic Bulletin & Review,**14*(3), 512–516. 10.3758/BF03194099 17874598 10.3758/bf03194099

[CR272] Satpute, A. B., & Lindquist, K. A. (2019). The default mode network’s role in discrete emotion. *Trends in Cognitive Sciences,**23*(10), 851–864. 10.1016/j.tics.2019.07.003 31427147 10.1016/j.tics.2019.07.003 PMC7281778

[CR273] Satpute, A. B., & Lindquist, K. A. (2021). At the neural intersection between language and emotion. *Affective Science,**2*(2), 207–220. 10.1007/s42761-021-00032-2 36043170 10.1007/s42761-021-00032-2 PMC9382959

[CR274] Saysani, A. (2019). How the blind hear colour. *Perception,**48*(3), 237–241. 10.1177/0301006619830940 30755088 10.1177/0301006619830940

[CR275] Saysani, A., Corballis, M. C., & Corballis, P. M. (2018). Colour envisioned: Concepts of colour in the blind and sighted. *Visual Cognition,**26*(5), 382–392.

[CR276] Saysani, A., Corballis, M. C., & Corballis, P. M. (2021). Seeing colour through language: Colour knowledge in the blind and sighted. *Visual Cognition,**29*(1), 63–71.

[CR277] Schifferstein, H. N., & Tanudjaja, I. (2004). Visualising fragrances through colours: The mediating role of emotions. *Perception,**33*(10), 1249–1266. 15693669 10.1068/p5132

[CR278] Schilder, J. D., IJzerman, H., & Denissen, J. J. A. (2014). Physical warmth and perceptual focus: A replication of IJzerman and Semin (2009). *PLoS One,**9*(11), Article e112772. 10.1371/journal.pone.0112772 25402343 10.1371/journal.pone.0112772 PMC4234632

[CR279] Schnall, S., Haidt, J., Clore, G. L., & Jordan, A. H. (2008). Disgust as embodied moral judgment. *Personality and Social Psychology Bulletin,**34*(8), 1096–1109. 10.1177/0146167208317771 18505801 10.1177/0146167208317771 PMC2562923

[CR280] Schoel, C., Eck, J., & Greifeneder, R. (2014). A matter of vertical position: Consequences of ostracism differ for those above versus below its perpetrators. *Social Psychological and Personality Science,**5*(2), 149–157. 10.1177/1948550613488953

[CR281] Schubert, T. W. (2005). Your highness: Vertical positions as perceptual symbols of power. *Journal of Personality and Social Psychology,**89*(1), 1–21. 10.1037/0022-3514.89.1.1 16060739 10.1037/0022-3514.89.1.1

[CR282] Seo, H.-S., Arshamian, A., Schemmer, K., Scheer, I., Sander, T., Ritter, G., & Hummel, T. (2010). Cross-modal integration between odors and abstract symbols. *Neuroscience Letters,**478*(3), 175–178. 10.1016/j.neulet.2010.05.011 20470860 10.1016/j.neulet.2010.05.011

[CR283] Seo, H.-S., Lohse, F., Luckett, C. R., & Hummel, T. (2014). Congruent sound can modulate odor pleasantness. *Chemical Senses,**39*(3), 215–228. 10.1093/chemse/bjt070 24368256 10.1093/chemse/bjt070

[CR284] Seymour, P. H. (1974). Asymmetries in judgments of verticality. *Journal of Experimental Psychology,**102*(3), 447–455. 10.1037/h0035865 4815191 10.1037/h0035865

[CR285] Shaki, S., & Fischer, M. H. (2008). Reading space into numbers—A cross-linguistic comparison of the SNARC effect. *Cognition,**108*(2), 590–599. 10.1016/j.cognition.2008.04.001 18514179 10.1016/j.cognition.2008.04.001

[CR286] Shepard, R. N. (1987). Toward a universal law of generalization for psychological science. *Science,**237*(4820), 1317–1323. 10.1126/science.3629243 3629243 10.1126/science.3629243

[CR287] Shepard, R. N. (1994). Perceptual-cognitive universals as reflections of the world. *Psychonomic Bulletin & Review,**1*(1), 2–28. 10.3758/BF03200759 24203412 10.3758/BF03200759

[CR288] Shepard, R. N., & Cooper, L. A. (1992). Representation of colors in the blind, color-blind, and normally sighted. *Psychological Science,**3*(2), 97–104.

[CR289] Sherman, G. D., & Clore, G. L. (2009). The color of sin: White and black are perceptual symbols of moral purity and pollution. *Psychological Science,**20*(8), 1019–1025. 10.1111/j.1467-9280.2009.02403.x 19619180 10.1111/j.1467-9280.2009.02403.x PMC2832478

[CR290] Shermer, D. Z., & Levitan, C. A. (2014). Red hot: The crossmodal effect of color intensity on perceived piquancy. *Multisensory Research,**27*(3/4), 207–223. 10.1163/22134808-00002457 25577903 10.1163/22134808-00002457

[CR291] Sidhu, D. M., & Pexman, P. M. (2018). Five mechanisms of sound symbolic association. *Psychonomic Bulletin & Review,**25*(5), 1619–1643. 28840520 10.3758/s13423-017-1361-1

[CR292] Sievers, B., Parkinson, C., Kohler, P. J., Hughes, J. M., Fogelson, S. V., & Wheatley, T. (2021). Visual and auditory brain areas share a representational structure that supports emotion perception. *Current Biology*. 10.1016/j.cub.2021.09.043 34644547 10.1016/j.cub.2021.09.043

[CR293] Sievers, B., Polansky, L., Casey, M., & Wheatley, T. (2013). Music and movement share a dynamic structure that supports universal expressions of emotion. *Proceedings of the National Academy of Sciences of the United States of America,**110*(1), 70–75. 10.1073/pnas.1209023110 23248314 10.1073/pnas.1209023110 PMC3538264

[CR294] Simms, N. K., & Gentner, D. (2019). Finding the middle: Spatial language and spatial reasoning. *Cognitive Development,**50*, 177–194. 10.1016/j.cogdev.2019.04.002

[CR295] Slepian, M. L., & Ambady, N. (2014). Simulating sensorimotor metaphors: Novel metaphors influence sensory judgments. *Cognition,**130*(3), 309–314. 10.1016/j.cognition.2013.11.006 24374210 10.1016/j.cognition.2013.11.006

[CR296] Slocombe, B. G., Carmichael, D. A., & Simner, J. (2016). Cross-modal tactile–taste interactions in food evaluations. *Neuropsychologia,**88*, 58–64. 10.1016/j.neuropsychologia.2015.07.011 26169315 10.1016/j.neuropsychologia.2015.07.011 PMC5072487

[CR297] Smith, L. B., & Sera, M. D. (1992). A developmental analysis of the polar structure of dimensions. *Cognitive Psychology,**24*(1), 99–142. 10.1016/0010-0285(92)90004-L 1537233 10.1016/0010-0285(92)90004-l

[CR298] Song, H., Vonasch, A. J., Meier, B. P., & Bargh, J. A. (2012). Brighten up: Smiles facilitate perceptual judgment of facial lightness. *Journal of Experimental Social Psychology,**48*(1), 450–452. 10.1016/j.jesp.2011.10.003

[CR299] Souter, N. E., Lindquist, K. A., & Jefferies, E. (2021). Impaired emotion perception and categorization in semantic aphasia. *Neuropsychologia,**162*, Article 108052. 10.1016/j.neuropsychologia.2021.108052 34624259 10.1016/j.neuropsychologia.2021.108052

[CR300] Speed, L. J., Atkinson, H., Wnuk, E., & Majid, A. (2021). The sound of smell: Associating odor valence with disgust sounds. *Cognitive Science,**45*(5), Article e12980. 10.1111/cogs.12980 34018230 10.1111/cogs.12980

[CR301] Speed, L. J., Croijmans, I., Dolscheid, S., & Majid, A. (2021). Crossmodal associations with olfactory, auditory, and tactile stimuli in children and adults. *I-Perception,**12*(6), 20416695211048510. 10.1177/20416695211048513 34900211 10.1177/20416695211048513 PMC8652194

[CR302] Speed, L. J., & Majid, A. (2018). Superior olfactory language and cognition in odor-color synaesthesia. *Journal of Experimental Psychology. Human Perception and Performance,**44*(3), 468–481. 10.1037/xhp0000469 28816480 10.1037/xhp0000469

[CR303] Spence, C. (2011). Crossmodal correspondences: A tutorial review. *Attention, Perception, & Psychophysics,**73*(4), 971–995. 10.3758/s13414-010-0073-7 10.3758/s13414-010-0073-7 21264748

[CR304] Spence, C., & Levitan, C. A. (2021). Explaining crossmodal correspondences between colours and tastes. *I-Perception,**12*(3), Article 20416695211018223. 10.1177/20416695211018223 34211685 10.1177/20416695211018223 PMC8216361

[CR305] Spence, C., & Parise, C. V. (2012). The cognitive neuroscience of crossmodal correspondences. *I-Perception,**3*(7), 410–412. 10.1068/i0540ic 23145291 10.1068/i0540ic PMC3485837

[CR306] Srinivasan, M., & Carey, S. (2010). The long and the short of it: On the nature and origin of functional overlap between representations of space and time. *Cognition,**116*(2), 217–241. 10.1016/j.cognition.2010.05.005 20537324 10.1016/j.cognition.2010.05.005 PMC2900540

[CR307] Steiner, J. E., Glaser, D., Hawilo, M. E., & Berridge, K. C. (2001). Comparative expression of hedonic impact: Affective reactions to taste by human infants and other primates. *Neuroscience and Biobehavioral Reviews,**25*(1), 53–74. 10.1016/S0149-7634(00)00051-8 11166078 10.1016/s0149-7634(00)00051-8

[CR308] Stellar, J. E., & Willer, R. (2014). The corruption of value: Negative moral associations diminish the value of money. *Social Psychological and Personality Science,**5*(1), 60–66. 10.1177/1948550613484770

[CR309] Stevens, J. C., & Marks, L. E. (1965). Cross-modality matching of brightness and loudness. *Proceedings of the National Academy of Sciences of the United States of America,**54*(2), 407–411. 5217429 10.1073/pnas.54.2.407 PMC219679

[CR310] Stevens, S. S. (1957). On the psychophysical law. *Psychological Review,**64*(3), 153–181. 13441853 10.1037/h0046162

[CR311] Stevenson, R. J., & Boakes, R. A. (2004). Sweet and sour smells: Learned synesthesia between the senses of taste and smell. In G. A. Calvert, C. Spence, & B. E. Stein (Eds.), *The handbook of multisensory processes* (pp. 69–83). Boston Review. 10.7551/mitpress/3422.001.0001

[CR312] Stevenson, R. J., Prescott, J., & Boakes, R. A. (1999). Confusing tastes and smells: How odours can influence the perception of sweet and sour tastes. *Chemical Senses,**24*(6), 627–635. 10.1093/chemse/24.6.627 10587495 10.1093/chemse/24.6.627

[CR313] Strickland, B., Aristodemo, V., Kuhn, J., & Geraci, C. (2017). The categorical role of structurally iconic signs. *Behavioral and Brain Sciences,**40*, Article E72. 10.1017/S0140525X15003076 29342537 10.1017/S0140525X15003076

[CR314] Strickland, B., Geraci, C., Chemla, E., Schlenker, P., Kelepir, M., & Pfau, R. (2015). Event representations constrain the structure of language: Sign language as a window into universally accessible linguistic biases. *Proceedings of the National Academy of Sciences of the United States of America,**112*(19), 5968–5973. 10.1073/pnas.1423080112 25918419 10.1073/pnas.1423080112 PMC4434776

[CR315] Thibodeau, P. H., & Boroditsky, L. (2011). Metaphors we think with: The role of metaphor in reasoning. *PLoS One,**6*(2), Article e16782. 21373643 10.1371/journal.pone.0016782 PMC3044156

[CR316] Tighe, L. S., & Tighe, T. J. (1966). Discrimination learning: Two views in historical perspective. *Psychological Bulletin,**66*(5), 353–370. 10.1037/h0023873 4859871 10.1037/h0023873

[CR317] Tonelli, A., Cuturi, L. F., & Gori, M. (2017). The influence of auditory information on visual size adaptation. *Frontiers in Neuroscience*. 10.3389/fnins.2017.00594 29114201 10.3389/fnins.2017.00594 PMC5660698

[CR318] Toomarian, E. Y., & Hubbard, E. M. (2018). On the genesis of spatial-numerical associations: Evolutionary and cultural factors co-construct the mental number line. *Neuroscience and Biobehavioral Reviews,**90*, 184–199. 10.1016/j.neubiorev.2018.04.010 29684402 10.1016/j.neubiorev.2018.04.010 PMC5993626

[CR319] Torralbo, A., Santiago, J., & Lupiáñez, J. (2006). Flexible conceptual projection of time onto spatial frames of reference. *Cognitive Science,**30*(4), 745–757. 10.1207/s15516709cog0000_67 21702834 10.1207/s15516709cog0000_67

[CR320] Tourangeau, R., & Sternberg, R. J. (1981). Aptness in metaphor. *Cognitive Psychology,**13*(1), 27–55.

[CR321] Tourangeau, R., & Sternberg, R. J. (1982). Understanding and appreciating metaphors. *Cognition,**11*(3), 203–244. 7199412 10.1016/0010-0277(82)90016-6

[CR322] Turner, S., & Littlemore, J. (2023). The many faces of creativity: Exploring synaesthesia through a metaphorical lens. *Elements in Cognitive Linguistics*. 10.1017/9781108974813

[CR323] Turoman, N., Velasco, C., Chen, Y.-C., Huang, P.-C., & Spence, C. (2018). Symmetry and its role in the crossmodal correspondence between shape and taste. *Attention, Perception, & Psychophysics,**80*(3), 738–751. 10.3758/s13414-017-1463-x 10.3758/s13414-017-1463-x 29260503

[CR324] Tversky, A. (1977). Features of similarity. *Psychological Review,**84*(4), 327.

[CR325] Tye, M. (1995). A representational theory of pains and their phenomenal character. *Philosophical Perspectives,**9*, 223–239. 10.2307/2214219

[CR326] Vallesi, A., Binns, M. A., & Shallice, T. (2008). An effect of spatial–temporal association of response codes: Understanding the cognitive representations of time. *Cognition,**107*(2), 501–527. 10.1016/j.cognition.2007.10.011 18076872 10.1016/j.cognition.2007.10.011

[CR327] van Paridon, J., Liu, Q., & Lupyan, G. (2021). How do blind people know that blue is cold? Distributional semantics encode color-adjective associations. *Proceedings of the Annual Meeting of the Cognitive Science Society, 43.*

[CR328] Velasco, C., Woods, A. T., Marks, L. E., Cheok, A. D., & Spence, C. (2016). The semantic basis of taste-shape associations. *PeerJ,**4*, Article e1644. 10.7717/peerj.1644 26966646 10.7717/peerj.1644 PMC4783761

[CR329] Vierck, E., & Kiesel, A. (2010). Congruency effects between number magnitude and response force. *Journal of Experimental Psychology: Learning, Memory, and Cognition,**36*(1), 204–209. 10.1037/a0018105 20053055 10.1037/a0018105

[CR330] Walker, L., Walker, P., & Francis, B. (2012). A common scheme for cross-sensory correspondences across stimulus domains. *Perception,**41*(10), 1186–1192. 10.1068/p7149 23469700 10.1068/p7149

[CR331] Walker, P. (2012). Cross-sensory correspondences and cross talk between dimensions of connotative meaning: Visual angularity is hard, high-pitched, and bright. *Attention, Perception & Psychophysics,**74*(8), 1792–1809. 10.3758/s13414-012-0341-9 10.3758/s13414-012-0341-9 22833281

[CR332] Walker, P. (2016). Cross-sensory correspondences: A theoretical framework and their relevance to music. *Psychomusicology: Music, Mind, and Brain,**26*(2), 103–116. 10.1037/pmu0000130

[CR333] Walker, P., Bremner, J. G., Lunghi, M., Dolscheid, S., D. Barba, B., & Simion, F. (2018). Newborns are sensitive to the correspondence between auditory pitch and visuospatial elevation. *Developmental Psychobiology,**60*(2), 216–223. 10.1002/dev.21603 29355921 10.1002/dev.21603

[CR334] Walker, P., Bremner, J. G., Mason, U., Spring, J., Mattock, K., Slater, A., & Johnson, S. P. (2010). Preverbal infants’ sensitivity to synaesthetic cross-modality correspondences. *Psychological Science,**21*(1), 21–25. 10.1177/0956797609354734 20424017 10.1177/0956797609354734

[CR335] Walker, P., Bremner, J. G., Mason, U., Spring, J., Mattock, K., Slater, A., & Johnson, S. P. (2014). Preverbal infants are sensitive to cross-sensory correspondences: Much ado about the null results of Lewkowicz and Minar (2014). *Psychological Science,**25*(3), 835–836. 10.1177/0956797613520170 24463556 10.1177/0956797613520170

[CR336] Walker, P., & Smith, S. (1985). Stroop interference based on the multimodal correlates of haptic size and auditory pitch. *Perception,**14*(6), 729–736. 10.1068/p140729 3837874 10.1068/p140729

[CR337] Walsh, V. (2003). A theory of magnitude: Common cortical metrics of time, space and quantity. *Trends in Cognitive Sciences,**7*(11), 483–488. 10.1016/j.tics.2003.09.002 14585444 10.1016/j.tics.2003.09.002

[CR338] Wan, X., Woods, A. T., van den Bosch, J. J. F., McKenzie, K. J., Velasco, C., & Spence, C. (2014). Cross-cultural differences in crossmodal correspondences between basic tastes and visual features. *Frontiers in Psychology*, *5*. https://www.frontiersin.org/article/10.3389/fpsyg.2014.01365 10.3389/fpsyg.2014.01365 PMC4259000 25538643

[CR339] Wang, Q., & Spence, C. (2017). The role of pitch and tempo in sound-temperature crossmodal correspondences. *Multisensory Research,**30*(3/5), 307–320. 10.1163/22134808-00002564 31287077 10.1163/22134808-00002564

[CR340] Wang, Q., Wang, S., & Spence, C. (2016). “Turn up the taste”: Assessing the role of taste intensity and emotion in mediating crossmodal correspondences between basic tastes and pitch. *Chemical Senses,**41*(4), 345–356. 10.1093/chemse/bjw007 26873934 10.1093/chemse/bjw007 PMC4840871

[CR341] Westbury, C., Hollis, G., Sidhu, D. M., & Pexman, P. M. (2018). Weighing up the evidence for sound symbolism: Distributional properties predict cue strength. *Journal of Memory and Language,**99*, 122–150.

[CR342] Whiteford, K. L., Schloss, K. B., Helwig, N. E., & Palmer, S. E. (2018). Color, music, and emotion: Bach to the blues. *I-Perception,**9*(6), 2041669518808535. 10.1177/2041669518808535 30479734 10.1177/2041669518808535 PMC6240980

[CR343] Wilkowski, B. M., Meier, B. P., Robinson, M. D., Carter, M. S., & Feltman, R. (2009). “Hot-headed” is more than an expression: The embodied representation of anger in terms of heat. *Emotion,**9*(4), 464–477. 10.1037/a0015764 19653767 10.1037/a0015764

[CR344] Willems, R. M., Labruna, L., D’Esposito, M., Ivry, R., & Casasanto, D. (2011). A functional role for the motor system in language understanding: Evidence from theta-burst transcranial magnetic stimulation. *Psychological Science,**22*(7), 849–854. 10.1177/0956797611412387 21705521 10.1177/0956797611412387

[CR345] Williams, L. E., & Bargh, J. A. (2008). Experiencing physical warmth promotes interpersonal warmth. *Science,**322*(5901), 606–607. 10.1126/science.1162548 18948544 10.1126/science.1162548 PMC2737341

[CR346] Williams, L. E., & Bargh, J. A. (2008). Keeping one’s distance: The influence of spatial distance cues on affect and evaluation. *Psychological Science,**19*(3), 302–308. 10.1111/j.1467-9280.2008.02084.x 18315805 10.1111/j.1467-9280.2008.02084.x PMC2394280

[CR347] Wilson, N. L., & Gibbs, R. (2007). Real and imagined body movement primes metaphor comprehension. *Cognitive Science,**31*(4), 721–731. 10.1080/15326900701399962 21635315 10.1080/15326900701399962

[CR348] Winkielman, P., & Cacioppo, J. T. (2001). Mind at ease puts a smile on the face: Psychophysiological evidence that processing facilitation elicits positive affect. *Journal of Personality and Social Psychology,**81*(6), 989–1000. 10.1037/0022-3514.81.6.989 11761320

[CR349] Winter, B. (2016). *The sensory structure of the English Lexicon.* UC Merced. https://escholarship.org/uc/item/885849k9

[CR350] Winter, B., Marghetis, T., & Matlock, T. (2015). Of magnitudes and metaphors: Explaining cognitive interactions between space, time, and number. *Cortex,**64*, 209–224. 10.1016/j.cortex.2014.10.015 25437376 10.1016/j.cortex.2014.10.015

[CR351] Woods, A. T., & Spence, C. (2016). Using single colors and color pairs to communicate basic tastes. *I-Perception,**7*(4), 2041669516658817. 10.1177/2041669516658817 27698979 10.1177/2041669516658817 PMC5030750

[CR352] Xu, A. J., & Labroo, A. A. (2014). Incandescent affect: Turning on the hot emotional system with bright light. *Journal of Consumer Psychology,**24*(2), 207–216. 10.1016/j.jcps.2013.12.007

[CR353] Yau, J. M., Olenczak, J. B., Dammann, J. F., & Bensmaia, S. J. (2009). Temporal frequency channels are linked across audition and touch. *Current Biology,**19*(7), 561–566. 10.1016/j.cub.2009.02.013 19268591 10.1016/j.cub.2009.02.013 PMC2700739

[CR354] Yehudai, A., Karidi, T., Stanovsky, G., Goldstein, A., & Abend, O. (2024, May 23). A nurse is blue and elephant is rugby: Cross domain alignment in large language models reveal human-like patterns. *ArXiv Preprints*. https://arxiv.org/abs/2405.14863v1

[CR355] Yuan, L., Uttal, D., & Gentner, D. (2017). Analogical processes in children’s understanding of spatial representations. *Developmental Psychology,**53*(6), 1098–1114. 10.1037/dev0000302 28358539 10.1037/dev0000302

[CR356] Yuan, T., Rau, P.-L., Zhao, J., & Zheng, J. (2023). Colour–touch cross-modal correspondence and its impact on single-modal judgement in multimodal perception. *Multisensory Research*(5). 10.1163/22134808-bja10098

[CR357] Zhao, Q., Ahrens, K., & Huang, C.-R. (2022). Linguistic synesthesia is metaphorical: A lexical-conceptual account. *Cognitive Linguistics,**33*(3), 553–583. 10.1515/cog-2021-0098

[CR358] Zhong, C.-B., & Leonardelli, G. J. (2008). Cold and lonely: Does social exclusion literally feel cold? *Psychological Science,**19*(9), 838–842. 10.1111/j.1467-9280.2008.02165.x 18947346 10.1111/j.1467-9280.2008.02165.x

[CR359] Zhong, C.-B., & Liljenquist, K. (2006). Washing away your sins: Threatened morality and physical cleansing. *Science,**313*, 1451–1452. 10.1126/science.1130726 16960010 10.1126/science.1130726

[CR360] Zhu, R., Goddu, M. K., Zhu, L. Z., & Gopnik, A. (2024). Preschoolers’ comprehension of functional metaphors. *Open Mind,**8*, 924–949. 10.1162/opmi_a_00152 39077109 10.1162/opmi_a_00152 PMC11285420

[CR361] Zizzo, D. J. (2010). Experimenter demand effects in economic experiments. *Experimental Economics,**13*(1), 75–98. 10.1007/s10683-009-9230-z

[CR362] Zohar-Shai, B., Tzelgov, J., Karni, A., & Rubinsten, O. (2017). It does exist! A left-to-right spatial-numerical association of response codes (SNARC) effect among native Hebrew speakers. *Journal of Experimental Psychology: Human Perception and Performance,**43*(4), 719–728. 10.1037/xhp0000336 28182477 10.1037/xhp0000336

